# Vegetation forcing modulates global land monsoon and water resources in a CO_2_-enriched climate

**DOI:** 10.1038/s41467-020-18992-7

**Published:** 2020-10-14

**Authors:** Jiangpeng Cui, Shilong Piao, Chris Huntingford, Xuhui Wang, Xu Lian, Amulya Chevuturi, Andrew G. Turner, Gabriel J. Kooperman

**Affiliations:** 1 Sino-French Institute for Earth System Science, College of Urban and Environmental Sciences, Peking University, 100871 Beijing, China; 2 Key Laboratory of Alpine Ecology and Biodiversity, Institute of Tibetan Plateau Research, Chinese Academy of Sciences, 100101 Beijing, China; 3 Center for Excellence in Tibetan Earth Science, Chinese Academy of Sciences, 100101 Beijing, China; 4 UK Centre for Ecology and Hydrology, Wallingford, Oxfordshire OX10 8BB UK; 5 National Centre for Atmospheric Science, University of Reading, Reading, RG6 6BB UK; 6 Department of Meteorology, University of Reading, Reading, RG6 6BB UK; 7 Department of Geography, University of Georgia, Athens, GA USA

**Keywords:** Atmospheric dynamics, Climate change, Hydrology, Hydrology

## Abstract

The global monsoon is characterised by transitions between pronounced dry and wet seasons, affecting food security for two-thirds of the world’s population. Rising atmospheric CO_2_ influences the terrestrial hydrological cycle through climate-radiative and vegetation-physiological forcings. How these two forcings affect the seasonal intensity and characteristics of monsoonal precipitation and runoff is poorly understood. Here we use four Earth System Models to show that in a CO_2_-enriched climate, radiative forcing changes drive annual precipitation increases for most monsoon regions. Further, vegetation feedbacks substantially affect annual precipitation in North and South America and Australia monsoon regions. In the dry season, runoff increases over most monsoon regions, due to stomatal closure-driven evapotranspiration reductions and associated atmospheric circulation change. Our results imply that flood risks may amplify in the wet season. However, the lengthening of the monsoon rainfall season and reduced evapotranspiration will shorten the water resources scarcity period for most monsoon regions.

## Abstract (web summary)

Monsoon systems have strong impacts on precipitation and food security over large areas of the world. Here, the authors show that plant responses to rising CO_2_ concentrations in the atmosphere play a key role in modulating seasonal rainfall and water resources over global land monsoon regions.

## Introduction

The global monsoon is a major feature of the Earth’s climate and is characterised by pronounced shifts between wet and dry seasons^1,2^. Any change in the seasonal distribution of precipitation can have far-reaching impacts on the availability of freshwater resources that support ecosystem sustainability^3^, agricultural production^1^ and the livelihood of two-thirds of the world’s population^4^. For instance, an increase in wet-season precipitation may amplify extreme precipitation and flood risk^4,5^, whereas a decrease in dry-season precipitation may exacerbate the scarcity of freshwater resources. Previous studies suggest an increase in precipitation in global monsoon systems, which may result from enhanced low-level moisture convergence due to the thermodynamic response to rising atmospheric CO_2_^6–8^. Additionally, weakening atmospheric dynamics may modulate the rates of precipitation increase in some monsoon regions^9^. However, detailed knowledge of monsoon seasonality and its response to global climate change is limited, as most previous studies focus only on expected seasonal mean rainfall changes^6–8^. Furthermore, few studies have investigated how the seasonal variation of water resources, and especially runoff, will change for global land monsoon regions. Runoff will be affected by both evolving rainfall characteristics and land-surface processes.

The Intergovernmental Panel on Climate Change (IPCC) confirms that increases in anthropogenic CO_2_ emissions are the primary driver of current climate change^10^. Rising atmospheric CO_2_ concentrations influence the terrestrial hydrological cycle by trapping longwave radiation (radiative forcing, RAD), which induces surface warming and associated changes in climate^11,12^. In addition, higher atmospheric CO_2_ levels can reduce stomatal conductance in plants and lead to lower transpiration rates per unit leaf area^13–19^, but simultaneously can also increase transpiration by expanding plant leaf area^20,21^ (vegetation-physiological forcing, VEG). Changes to RAD and VEG may contribute to reshaping the seasonality of surface water resources by altering either precipitation, or plant transpiration, or both. Therefore, accurate separation of the contribution of these two forcings is critical to understanding their respective roles in future seasonal changes of precipitation and water resources over global land monsoon regions^1,13,15,22,23^.

Here, we investigate the relative roles of VEG and RAD in driving the future hydrological cycle for seven large-scale land monsoon regions. We find that in a CO_2_-enriched climate, vegetation feedbacks strongly affect annual precipitation, and in particular for North and South America and Australia monsoon regions. Stomatal closure-driven evapotranspiration reductions and associated atmospheric circulation change increase dry season runoff and also shorten the water resources scarcity period for most monsoon regions. These findings highlight the importance of plant responses to rising CO_2_ for modulating monsoon projections and their potential buffering of global warming-induced water scarcity.

## Results

### Simulation structure

Our analysis of expected future changes are based predominantly on results from Coupled Model Intercomparison Project phase 5 (CMIP5) Earth System Model (ESM) simulations with 1% per year increases in atmospheric CO_2_ concentrations, which reach a quadrupling of preindustrial levels after 140 modelled years^24^. A subset of ESMs (Supplementary Table 1) performed this scenario, using idealised single-forcing simulations. These factorial experiments consider the influence of rising CO_2_ on physiological (VEG) or radiative (RAD) processes independently, in order to compare their separate contributions against changes simulated with both forcings together (ALL; see Methods section and Supplementary Table 2). This particular framework enables differentiation between RAD and VEG drivers, which we analyse in the context of changes to rainfall patterns at the annual and seasonal scales. As the total wet season precipitation is a function of the average intensity of precipitation and monsoon length, our seasonal-scale assessment includes changes to both characteristics of monsoon (onset and retreat dates, as well as duration). In addition to mean precipitation, we also consider changes to the length (start and end dates) of the period in which water resources, and notably runoff are abundant (Methods section). The performances of the ESMs we use are verified by observations at both the field scale (Supplementary Fig. 1) and the regional scale (Supplementary Fig. 2) for historical CO_2_ levels. We also compare against the same set of models when forced by representative concentration pathway 8.5 (RCP8.5; Supplementary Figs. 3 and 4) and, importantly, a cloud-resolving model SP-CESM1^25^ (see Methods section and Supplementary Figs. 5–8).

### Drivers of annual mean changes in monsoon rainfall and runoff

**Fig. 1:**
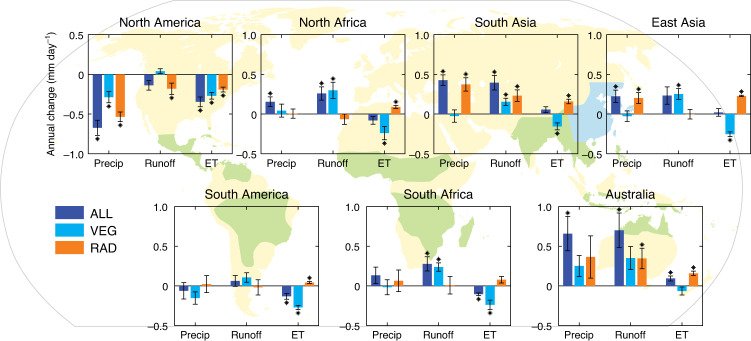
Annual-mean changes in precipitation (precip), runoff and evapotranspiration (ET) in different simulations. The dark blue, light blue and orange bars indicate the predictions from all CO_2_-based forcings (ALL), physiological only (VEG) and radiative only (RAD), respectively, on hydrological impacts and in each monsoon region as labelled. The bars are model mean, and the error bars (thin lines) indicate standard errors of the mean of the four ESMs analysed. Changes are quantified by the differences of the model years 121–140 (~4 × CO_2_-enriched), and the years 1–20 (historical; see Methods section). Asterisks indicate that the change is significant for the four ESMs (*P* < 0.05, *t*-test), and so the latter test is based on the standard errors to assess if there is a mean change. Regional monsoon domains are shown in light green colours on the background map, while East Asia is in light blue in order to distinguish it from South Asia.

At the annual scale, we find that for most monsoon regions, more water is available in a CO_2_-enriched climate. Annual precipitation increases in five out of the seven land monsoon regions (Fig. 1, dark blue bars), which is consistent with previous findings^6–8^. Precipitation does, however, decrease for North and South America, where the VEG forcing has a strong negative impact (ratios of VEG to total annual precipitation change in ALL: 0.43 and 2.50 for North and South America, respectively). In particular, as VEG is the dominant component for precipitation change in South America (Fig. 1), this highlights strong land-atmosphere feedbacks on future rainfall in the region. However, in South and East Asia, and North and South Africa monsoon regions, VEG-induced ET reduction has a limited impact on precipitation change, but contributes significantly to runoff increases. For the Australia monsoon region, VEG-induced ET decrease is small. The large precipitation increase in the VEG simulation (41%) for Australia is, therefore, likely to be an indirect effect of ET reduction elsewhere, and so via atmospheric circulation changes (see below). These results indicate that the mechanisms of vegetation feedbacks on regional precipitation change are substantial, yet quite distinct over different monsoon regions.

For runoff (Fig. 1), there is an increase in all land monsoon regions except North America, and notably in other locations, the magnitude of the runoff increase is equal to or larger than that of precipitation. In North and South Africa, South America and East Asia, a major contribution to the runoff enhancement is driven by a decrease in evapotranspiration (ET) resulting from reduced stomatal conductance with higher CO_2_ (VEG), which increases water flowing into rivers^26^. In particular for East Asia, RAD has no impact on runoff change since the increase in precipitation in RAD is almost completely offset by an increase in ET (i.e., increased evaporative demand with warming), so that runoff changes are controlled entirely by VEG. In the South Asia and Australia monsoon regions, both RAD and VEG forcings provide positive and comparable increases in runoff, giving an overall change that is even more substantial than in other regions. Overall, although RAD is the dominant component of precipitation change in most regions, the VEG forcing has a larger influence on runoff and ET, except for North America, South Asia (runoff only) and Australia (ET only). Some regions contain nonlinearities arising from interactions between RAD and VEG, as noted by the sum of RAD and VEG being sometimes different from ALL – e.g. for precipitation in North Africa.

### Drivers of seasonal changes in rainfall and runoff intensity

**Fig. 2:**
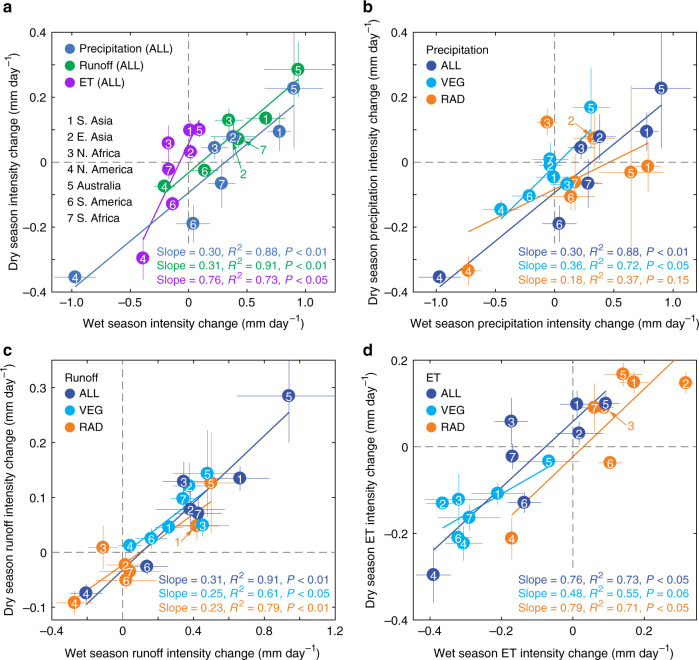
Intensity changes in precipitation, runoff and evapotranspiration (ET) and their drivers in wet and dry seasons. **a** Intensity change in precipitation, runoff and ET in wet (horizontal axis) and dry seasons (vertical axis) in all CO_2_-based forcings (ALL) simulations. Regions labelled as 1–7. Any overlapping circles are pointed to by labels connected by an arrow. The colour lines indicate the linear regressions between water cycle component changes in wet and dry seasons across the seven monsoon regions, with the slope value and statistical information shown in the bottom right. **b**–**d** Intensity changes in ALL simulations are further divided into physiological (RAD) and radiative (VEG) drivers in wet and dry seasons for **b** Precipitation, **c** Runoff, and **d** ET. Intensity changes are quantified by the differences of the years 121–140 (approximately atmospheric enrichment of 4 × CO_2_ of preindustrial levels) of simulations and the years 1–20 (historical) in the wet or dry seasons. The wet season is defined as the months where ET < precipitation, and vice versa for the dry season. Wet and dry season lengths are kept constant and correspond to those for the historical period and for each monsoon region. This approach allows changes in average seasonal intensity to be isolated from changes in season length (see Methods section). The thin lines are error bars, indicating the standard errors of the mean of the four ESMs.

In general, precipitation increases at the annual scale, but we find distinct precipitation intensity changes between wet and dry seasons. Intensity is defined as the mean seasonal precipitation rate over a fixed historical season length (see Methods for details of definitions). As shown in Fig. 2a, increased precipitation intensity mainly occurs in the wet season and in most monsoon regions, noting the larger horizontal-axis range. Projected ALL-forcings wet season increases are Australia: +0.90 mm day^−1^ or +15.4%, South Asia: +0.78 mm day^−1^ or +12.7%, East Asia: +0.38 mm day^−1^ or +7.6%, South Africa: +0.28 mm day^−1^ or +4.8%, North Africa: +0.22 mm day^−1^ or +4.7% and South America: +0.04 mm day^−1^ or +0.6%. The exception to these increases is North America (−0.97 mm day^−1^ or −20.6%), where dry season precipitation intensity also decreases. The decline of wet season precipitation in the North America monsoon region in response to rising CO_2_ is also projected by a previous study^27^. In contrast to larger wet season changes, the smaller dry season precipitation intensity changes (Fig. 2a) are increases in Australia (+0.23 mm day^−1^ or +12.2%), South Asia (+0.09 mm day^−1^ or +8.2%), East Asia (+0.08 mm day^−1^ or +4.3%) and North Africa (+0.04 mm day^−1^ or +5.5%), and decreases in North (−0.35 mm day^−1^ or −28.4%) and South America (−0.19 mm day^−1^ or −19.9%) and South Africa (−0.07 mm day^−1^ or −7.0%). Overall, although dry season precipitation intensity changes are less than those of wet seasons, the relative changes are larger than the wet season changes in North and South America, and North and South Africa. In addition, the positive linear relationships (colour best-fit lines in Fig. 2) between changes in wet and dry seasons and across the seven monsoon regions indicate that the intensity changes covary between wet and dry seasons for precipitation, runoff and evapotranspiration, and in all simulations (Fig. 2a, ALL simulations). For example, the regions with 1.00 mm day^−1^ greater precipitation or runoff increase in the wet season correspond to 0.30 mm day^−1^ and 0.31 mm day^−1^ greater increase in precipitation or runoff respectively in the dry season (Fig. 2a). As dry season precipitation intensity increases for some of the major monsoon regions, the hypothesis of “wet season-gets-wetter, dry season-gets-drier” proposed at global scales^28^, may not hold when characterising wet and dry seasonal regional variations under CO_2_-enriched climate.

Further analysis based on the single-forcing simulations, differentiated by wet or dry season, clarifies the contribution from RAD and VEG to seasonal precipitation intensity changes across different monsoon regions (Fig. 2b). As for annual precipitation change, RAD in general provides the dominant role in seasonal precipitation intensity change. For wet-season precipitation intensity change in the North Africa monsoon region, there is no clear dominant driver due to strong nonlinear interactions between VEG and RAD forcings. As a major exception, the dry-season precipitation intensity change in the Australia monsoon region is instead dominated by VEG (Fig. 2b). VEG and RAD both contribute equally to dry season precipitation changes in South America and offset each other for wet season changes. Furthermore, with the exception of East Asia (precipitation change in VEG simulation: −0.7% in wet season and −0.6% in dry season) and South Africa (−0.6% in wet season and 1.0% in dry season), the magnitude of VEG-induced precipitation change is substantial in at least one season across all regions. The range for the other locations is −0.45 to +0.31 mm day^−1^ (−9.6% to +5.3%) in the wet season and −0.15 to +0.17 mm day^−1^ (−11.4% to 9.2%) in the dry season; as measured by the span of light blue circle distances from the origin (0,0) point in Fig. 2b. These values reiterate the important role of vegetation-atmosphere feedbacks on monsoon dynamics, and here at the seasonal timescale.

**Fig. 3:**
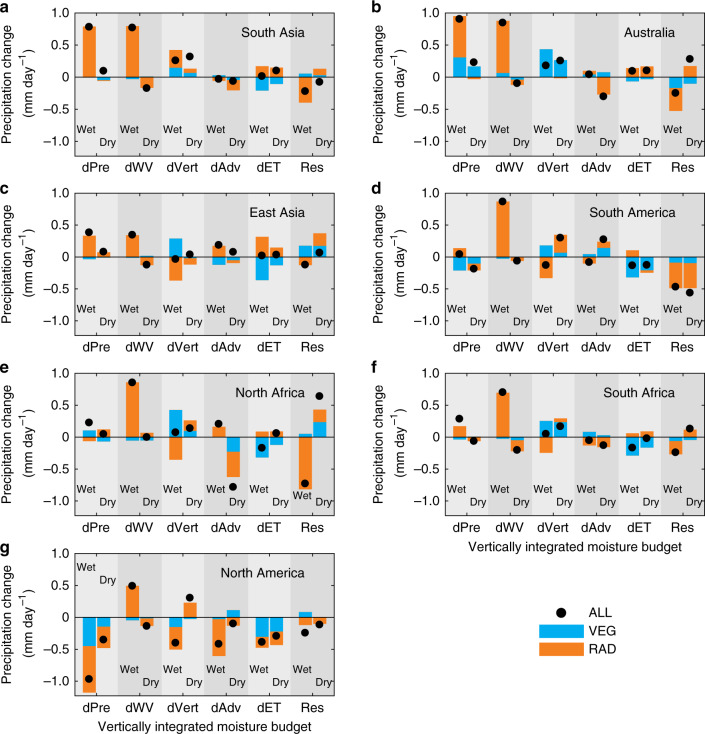
Regional components of precipitation change associated with different processes in both wet and dry seasons. Each panel **a**–**g** corresponds to one of seven monsoon regions, as marked. Monsoon regions on the left-hand and right-hand sides are located in Northern Hemisphere and Southern Hemisphere, respectively. In each panel, different contributions to rainfall changes are presented. The first column pair are the total precipitation change (dPre). The next four column pairs are: thermodynamic effect, i.e., atmospheric water vapour changes (dWV), vertical dynamic effect changes (dVert), altered horizontal advection (dAdv) and land evapotranspiration change (dET), respectively (see Methods section). The Residual effect (Res) is the difference between total precipitation change (dPre) and the sum of the four processes (dWV + dVert + dAdv + dET). For dPre, the four individual drivers and Res, there is a further subdivision, with the left and right columns showing changes in the wet and dry seasons respectively. Each process is further divided into radiative (RAD, orange bars) and physiological (VEG, blue bars) drivers in wet and dry seasons, while the black circles are from the ALL simulations.

**Fig. 4:**
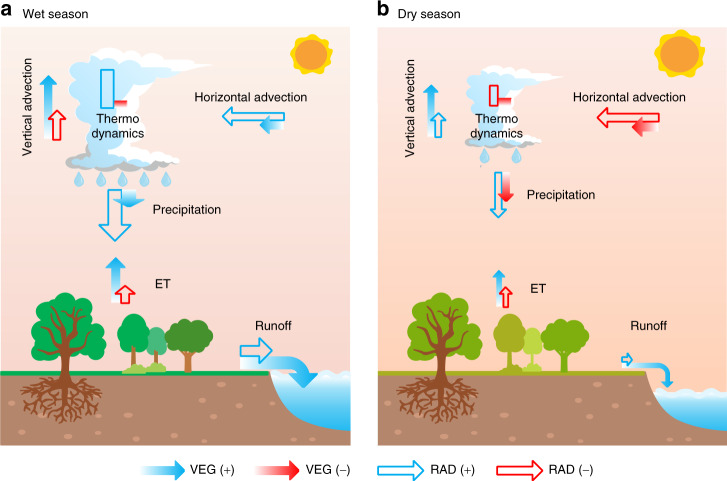
Schematic diagram of the mechanisms driving seasonal monsoon precipitation and runoff changes in the five monsoon regions with annual precipitation increase. Changes in precipitation, runoff and evapotranspiration (ET) in **a** wet and **b** dry seasons. Precipitation change is divided into thermodynamic effect, vertical advection, horizontal advection and land ET components as marked (see also Methods section). The solid and open arrows (or rectangles) represent the vegetation-physiological (VEG) and radiative forcings (RAD) of rising CO_2_ in each process, respectively. The blue or red colour indicates if the change is beneficial (+) or adverse (−) to runoff change, respectively, rather than the sign of the change itself. The size of the arrows (or rectangles) denotes the relative magnitude of each change averaged over the five monsoon regions (hence excluding North and South America). The signs of precipitation and its component changes are generally consistent among the five monsoon regions, although there are a small number of exceptions (Supplementary Table 3). In the wet season **a**, RAD-induced warming dominates the increases in atmospheric precipitable water, advection and precipitation, although VEG forcing via rising CO_2_ also increases precipitation by enhancing atmospheric circulation (vertical advection and horizontal advection). VEG-induced land ET reduction and precipitation increase result in more water flowing as runoff. In the dry season **b**, RAD-induced increases in vertical advection and land ET overcome the negative impacts of horizontal advection and thermodynamic decreases, facilitating precipitation increase, although VEG exerts an adverse impact on precipitation. However, VEG-induced ET decrease still drives an increase in runoff.

The VEG and RAD forcings from higher atmospheric CO_2_ affect precipitation through four processes: thermodynamic effects, represented by atmospheric water vapour changes (dWV), as well as altered atmospheric vertical advection (dVert), altered horizontal advection (dAdv) and land-surface ET changes (dET)^29–32^. For both the wet and dry seasons, and for each region, we decompose (see Methods section) precipitation intensity (dPre) into these four processes^31^ (Fig. 3 and schematic Fig. 4). The difference between precipitation intensity and the sum of the four processes is marked as the residual term (Fig. 3, Res). Figure 3 reveals that the processes driving precipitation intensity changes in wet and dry seasons are distinct. Precipitation intensity increases in the wet season are often dominated by thermodynamic effects, due to large positive values of dWV in all monsoon regions. The exception is North America, where instead the significant precipitation reduction is driven by RAD-induced circulation weakening and land ET decrease (Fig. 3g). In the dry season, however, rainfall changes are dominated by combinations of the other three components of (dET, dAdv and dVert). It is noted that the residual components, that include transient eddies (storm systems) and nonlinear cross-terms, are much larger in South America and North Africa than elsewhere. This large residual effect indicates a low confidence of precipitation change decomposition in these regions. The inclusion of transient eddies in the residual term may amplify the uncertainties of decomposition, although a previous study suggested that the impact of transient eddies on precipitation change is small compared with other components and for all monsoon regions^9^.

As further evidence of land-surface feedbacks on the atmosphere above, we find that VEG effects play critical roles in driving atmospheric circulation change (dAdv+dVert) in both wet and dry seasons across all monsoon regions (Fig.3). Of particular interest is that in the five regions where precipitation is projected to increase (excluding North and South America monsoon regions), on average VEG dominates vertical advection change in both wet and dry seasons (Figs. 3 and4). In the wet season, VEG increases vertical gradients of both air temperature and specific humidity (Supplementary Fig. 9), and thus facilitates upward transport of moisture by convection, initiating precipitation increases (Figs. 3 and 4a). In the dry season, the changes in specific humidity are generally small compared to those in the wet season. However, VEG does still strengthen the air temperature gradient through low-level heating in the dry season, which will again enhance convection and hence precipitation (Supplementary Fig. 9 and Fig. 4b). The influence of VEG forcing on air temperature and specific humidity in the lower troposphere and its links to seasonal precipitation may be associated with atmospheric circulation and moisture convergence changes^15,33^, which requires further study. These gradient changes are projected as especially strong in Australia (wet and dry seasons) and South Africa (dry season) monsoon regions, as suggested by our finding that vertical advection is almost completely controlled by VEG in both regions (Fig. 3b, f). To date, most existing studies have focused only on the impacts of RAD forcing on atmospheric circulation and subsequent precipitation^12,34^, though our accounting for VEG-based drivers supports the role of vegetation-based forcing, and as is beginning to gain attention^15,33,35^. Yet biases in atmospheric circulation remain the major source of uncertainty in current precipitation projections^36^. Hence further work to constrain the parameterisation of terrestrial ecosystem models will likely help towards lowering uncertainties in simulating atmospheric circulations^33,35^ and related monsoon precipitation projections.

In addition to precipitation, we also analyse runoff changes, as this is an important impact for society, affecting freshwater availability and flooding. Similar to precipitation, the projected wet season runoff changes are larger than those of the dry season (Fig. 2c). Also, in the ALL simulations with raised atmospheric CO_2_, runoff intensity increases are generally larger than precipitation intensity increases in the wet season, implying that the land response under rising CO_2_ may further amplify wet-season flood risks over most monsoon regions. In the dry season, we find that runoff generally increases over most monsoon regions (except North and South America; Fig. 2c). Critically, for locations where precipitation intensity is projected to decrease in the dry season, runoff intensity decreases will be smaller. This finding for the dry season is because the land surface will offset the negative impacts of precipitation decreases as CO_2_ rises, largely due to a decline in stomatal conductance that reduces land-surface ET. However, in the Australia monsoon region, VEG-induced runoff increase in the dry season is instead largely contributed by a land-surface feedback causing precipitation increase, with direct ET reduction plays a secondary role. Indeed, VEG effects dominate runoff intensity increases in both wet and dry seasons for most of the monsoon regions except South Asia, North America, Australia (wet season) and South America (dry season; Fig. 2c). This result is not in conflict with the previous reporting that rising atmospheric CO_2_ concentrations may have had little impact on annual runoff changes in the last century^37^. Although initial increases in leaf area index (LAI) in response to rising CO_2_ concentration over the 20th century^20^ and in the past 30 years^38^ may raise ET, the rate of LAI increase slows down at higher levels of CO_2_. Hence at quadruple preindustrial CO_2_ levels^20^, stomatal closure may substantially overtake any fertilisation effects (Supplementary Fig. 10), explaining our simulated annual runoff increases.

### Mechanisms driving changes in seasonal characteristics

**Fig. 5:**
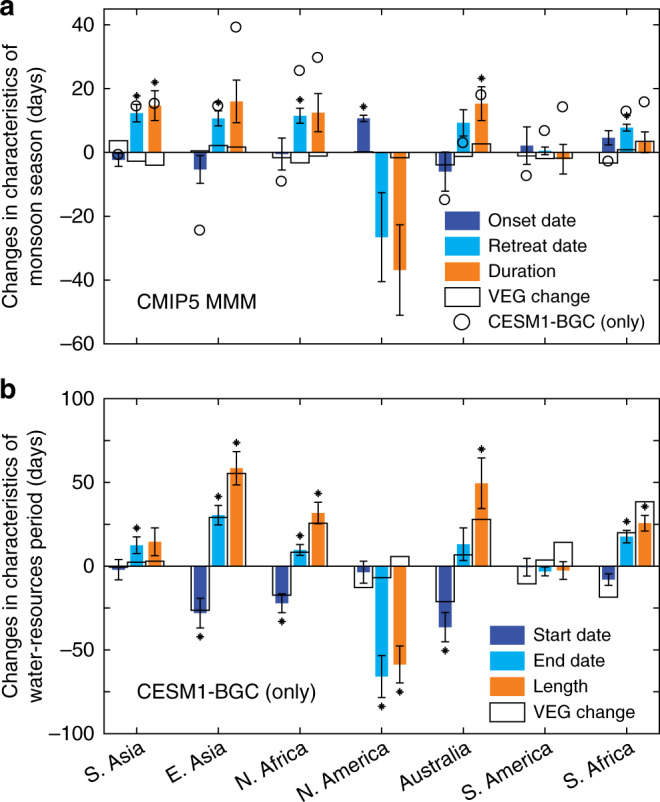
Changes in seasonal characteristics of monsoon season and abundant water-resources period. **a** Monsoon season changes from the CMIP5 multi-model mean (MMM) for the seven regions as marked. Indices of seasonal characteristics are monsoon onset date, retreat date and monsoon season duration, presented for the all CO_2_-based forcings (ALL) simulations (colour bars). The error bars indicate standard errors of the mean of the three ESMs. Small black asterisks indicate if changes are significant for the three ESMs (*P* < 0.05, *t*-test). The NorESM1-ME model is not included due to a lack of daily precipitation output in the CMIP5 archives. Black open rectangles indicate the changes associated with the physiological (VEG)-only forcing. Changes in monsoon season duration in ALL simulations from the CESM1-BGC only model are also presented (open circles). The changes in North America from CESM1-BGC are not available due to model dry bias (Supplementary Fig. 2d) and significant precipitation reductions at 4 × CO_2_ (Supplementary Fig. 11d). **b** Abundant water resources period. Seasonal characteristic indices are start date, end date and length of abundant water resources period. Values in **b** are for only one model, CESM1-BGC, as no daily runoff output is available in the CMIP5 archives for the four ESMs (see Methods section). The error bars indicate standard errors based on interannual variability of runoff from CESM1-BGC only. Small black asterisks indicate that the change is significant in the CO_2_-enriched climate relative to the preindustrial period (*P* < 0.05, *t*-test).

Since changes in both total monsoon precipitation and runoff may result from changes in intensity and length, we further investigate adjustments to the seasonal characteristics of the monsoon. Onset and retreat dates are defined as when daily precipitation initially exceeds or finally drops below a specific threshold^7,39^ (see Methods section). The difference between these dates, and how it changes, defines monsoon length changes for a CO_2_-enriched climate. We find a similar pattern of changes in monsoon season length (orange bars, Fig. 5a) with that of changes in annual precipitation across the seven monsoon regions (Fig. 1). North and South American monsoons have decreases in length of monsoon season of −37 and −2 days respectively, whereas lengths increase in the five other regions, generally supporting previous results^7,8^. The shortening of monsoon season length is driven by both delay to the onset date (+11 days) and advance of the retreat date (−26 days) for North America, and by delay to the monsoon onset date (+2 days) in South America (Fig. 5a). The extensions of season length over other monsoon regions, which vary from +3 days in South Africa to +16 days in East Asia, are instead mainly driven by delayed retreat dates (Fig. 5a). Our single-forcing simulations show that VEG extends monsoon season length in Australia (+3 days), South Africa (+3 days) and East Asia (+2 days) but has a negative contribution in other regions (black rectangles, Fig. 5a). Although the impacts of RAD are generally much more important than VEG in the changes of both onset and retreat dates across all monsoon regions, VEG effects are confirmed as playing an important role. The changes of onset dates (−4 to +4 days) and retreat dates (−3 to +2 days) in the VEG simulations further illustrate the substantial contribution made by physiological forcing on rainfall patterns.

We also explore wet and dry season length changes under the CO_2_-enriched climate, as defined by features of daily projected runoff. Similar to precipitation, we use the first day exceeding and the last day dropping below a runoff threshold as our main proxy for water abundance changes. The baseline thresholds are calculated from only one model, CESM1-BGC^40^, because no daily runoff output is available in the CMIP5 archives for our four ESMs (please see Methods for validation of single-model use). As expected, the length changes of the abundant runoff-based water-resources period (orange bars in Fig. 5b) show similar patterns to precipitation-based monsoon season length changes (open circles in Fig. 5a). For example, the abundant water-resources period increases in South and East Asia, Australia, and North and South Africa, and decrease in North America are close to those of the monsoon season duration. In Australia, however, the increase in length of abundant water resources (50 days) is about three times that of monsoon season length extension (18 days), while the change in length of abundant water resources is small (−3 days) compared to monsoon season length change (14 days) in the South America monsoon region. The extension of the abundant water resources period is generally caused by both earlier start and later end dates (Fig. 5b). The exception is North and South America, where the shortening of the abundant water-resources period is dominated by an earlier end date (−66 days in North America and −3 days in South America). Among the five monsoon regions with the longer abundant water resources period, the most substantial earlier change of start date is Australia (−36 days), and the largest delayed end date is East Asia (31 days). For both these locations, this generates overall annual runoff increases (Supplementary Fig. 11a, c). The changes in water-resources period are consistent with the substantial region-dependent increases or decreases of annual runoff in a CO_2_-enriched climate (Supplementary Fig. 11).

In most regions, the individual vegetation-based VEG forcing (black open rectangles in Fig. 5b) exerts a more substantial impact on changes to the features of the abundant water-resources period than the overall monsoon characteristics that includes RAD drivers. For example, East Asia, Australia, North Africa and South Africa all have large future extensions (26–59 days) of abundant water resources related to large VEG-induced impacts under a CO_2_-enriched climate (ratio of VEG to ALL: 1.5 for South Africa, 0.95 for East Asia, 0.81 for North Africa and 0.56 for Australia; Fig. 5b). The extent to which these expanded periods are due to changes in start and end dates is, however, location-specific. For instance, VEG extends the length of the abundant water resources period more by advancing the start date in Australia and North Africa. Instead, this period is extended more by delaying the end date in South Asia monsoon regions. In East Asia and South Africa monsoon regions, VEG exerts a comparable impact on advance of start date and delay to end date. The shortening of the abundant water-resource period in the North America monsoon region is mainly due to RAD (ratio of RAD to ALL: 1.1) compared to a much smaller impact from VEG (ratio of VEG to ALL: −0.1).

## Discussion

It is known that some ESMs may over- or underestimate precipitation in specific monsoon regions. For example, the modelled seasonal precipitation of the North America monsoon region is, overall, underestimated compared to observational precipitation and for the historical period (Supplementary Fig. 2). Any bias may affect the projection of changes in precipitation intensity and seasonal characteristics (monsoon onset, retreat dates and duration)^41,42^ in response to rising CO_2_. To check, we test whether perturbing our thresholds that characterise the monsoon period alters our results, and we find that our findings are generally not sensitive to different precipitation thresholds used (Supplementary Fig. 12). Furthermore, our estimates are also broadly consistent when instead using an independent cumulative-based index^43^ (Supplementary Fig. 13). This robustness to threshold definition implies that ESM-based estimates of monsoon changes can be used with confidence. The majority of the work presented here is on differences between historical and potential future climatic states (for 4×CO_2_), although further analysis could specifically bias-correct based on our metrics of monsoon seasonality, and for the historical period^44,45^.

In addition, ESMs can perform poorly in the simulation of the seasonal cycle of LAI. We test the mean of monthly modelled LAI from our ESMs and compare against standard MODIS C6 measurements (Supplementary Fig. 14). We find that our models are up to 2 months out of phase with observations, and particularly in the tropical monsoon regions. However, despite this, the modelled seasonality of ET and gross primary productivity (GPP) are generally consistent with observations (comparison against GLEAM v3.3a and MTE upscaling, respectively; Supplementary Fig. 14). Nevertheless, with our noted strong VEG impact on monsoon features, a better representation of vegetation phenology in land-surface models is desirable. Other authors note the importance of further improving modelled vegetation feedbacks on regional hydrological cycles and climate systems^46^. It is noted, though, that as stomatal closure dominates ET change, compared to the effects of LAI and under very high CO_2_ levels (Supplementary Fig. 10c), modelled LAI deficiencies may exert a limited impact on precipitation and runoff changes in our study.

Due to the limited availability of single-forcing factorial experiments, only four ESMs are currently available for analysis. Yet, although we expect our findings to be generally consistent, there are differences between the four climate models. Some consistency is expected in projections of transpiration, as a commonality between many simulations is the Ball-Berry stomatal conductance scheme (Supplementary Table 1). However, for each ESM, the land scheme likely has a different parameterisation (e.g. for LAI). Refined descriptions of land-atmosphere water exchange are needed. A recent study^47^ finds that current ESMs strongly underestimate the ratio of plant transpiration to total terrestrial evapotranspiration, which may influence the timings of VEG impacts on the global hydrological cycle. Hence, future efforts to better simulate plant physiological responses to rising CO_2,_ when implemented in coupled frameworks, will enhance the modelling of vegetation-atmosphere feedbacks and their roles in modifying monsoon rainfall features in both future and paleo climates. Such gains from improved land-surface models are additional to aiding refinement of estimates of future flood risk and water-resource availability.

Further verification is required for our model-based results, acknowledging some current model deficiencies in dry bias, seasonality and convection-driven precipitation. However, despite remaining uncertainties, we anticipate our findings to be broadly robust. In summary, we project an increase in precipitation over most global land monsoon regions in response to significantly raised atmospheric CO_2_ concentrations. In regions of raised intensity, the monsoon season length will also be extended, and in particular through delayed retreat dates. Although the changes in monsoon intensity are mainly driven by radiative forcing, particularly novel to our assessment is the discovery that physiological forcing effects can also be substantial. Our analysis therefore highlights the critical role of vegetation-atmosphere feedbacks in controlling monsoon attributes. For the key impact of runoff intensity change, the physiological impact of high CO_2_ levels is generally even larger than that of raised atmospheric CO_2_ altering radiative forcing, and thus climate. Vegetation response is therefore expected to amplify wet-season flood risks. However, more beneficially, the impact of CO_2_ on vegetation not only mitigates land-surface water scarcity in the dry season over monsoon regions but also extends the length of the abundant water-resources period.

## Methods

### Single-forcing model selection and data processing

We use six ESMs from the CMIP5 database^24^ (https://pcmdi.llnl.gov/?cmip5) for our assessment. These have the variables necessary for our analysis, correspond to the 1pctCO_2_ scenario (i.e 1% per year increase in atmospheric CO_2_ concentration) and, notably, have the appropriate single-forcing simulations. With our emphasis on land-atmosphere feedbacks in the hydrological cycle, we require ESMs to model the three components of evapotranspiration accurately. We find that there is a significant relationship between the ratio of annual mean transpiration to evapotranspiration (i.e. T/ET) of each ESM, and its sensitivity of terrestrial water cycle components to rising CO_2_ (Supplementary Fig. 15). ESMs that underestimate observed T/ET values have low sensitivity of transpiration to climate forcing from radiation and physiological responses to higher CO_2_ (Supplementary Fig. 15b, c). Previous research confirms that the inaccuracy of T/ET influences the modelling of terrestrial water cycles^47,48^ and biophysical feedbacks^49^. We therefore use estimates of land T/ET values from isotope observations^50^ and a range of these values within ±1 standard deviation to filter the six ESMs (Supplementary Fig. 15a). This gives four ESMs (CESM1-BGC, IPSL-CM5A-LR, MPI-ESM-LR and NorESM1-ME) that have values within these bounds, and so these were selected for analysis (Supplementary Table 1).

The four retained ESMs all contributed simulations to the three idealised experiments. All correspond to increasing CO_2_ concentration from preindustrial levels (285 ppm) to a quadrupling (1140 ppm) of preindustrial conditions. The CO_2_ concentration changes at a rate of 1% per year continuously, named 1pctCO_2_, over a period of 140 model years (Supplementary Table 2). Critically, ESM experiments have been performed in which rising CO_2_ concentrations influence the atmospheric model only (named RAD, esmFdbk1 in CMIP5 terminology), the land-surface model only (named VEG, esmFixClim1 in CMIP5 terminology), or both (named ALL, 1pctCO_2_ in CMIP5 terminology), as described in Arora et al.^51^. Monthly ESM diagnostics of precipitation, runoff and evapotranspiration are used to calculate annual and seasonal precipitation and runoff intensity change. Monthly specific humidity and air temperature allow investigation of the atmospheric dynamic processes in VEG simulations. Transpiration in VEG simulations is further partitioned into the parts caused by stomatal closure (stomata) and CO_2_ fertilisation on vegetation structure (LAI) through the annual changes in transpiration and LAI (Supplementary Fig. 10c). Daily precipitation and runoff are used to more precisely determine the response of seasonal characteristics of the monsoon and abundant water-resource periods (Supplementary Table 1). Summary statistics of the simulated changes are dervied from the difference between the average of the last 20 years (mean of 1036 ppm CO_2_, CO_2_-enriched) and the first 20 years (314 ppm CO_2_, historical). These statistics are calculated as the change, due to rising CO_2_, for all major water component variables and seasonal characteristics. The ratio of VEG change to total change in ALL simulation is used to determine the impact of VEG on each component or other variables. Ratios are used to eliminate the possible presence of compensating opposite signs between VEG and RAD which would otherwise obscure changes.

### CMIP5 RCP8.5 simulations

To assess the ability of our ESMs to capture reasonable precipitation, runoff and ET responses to rising CO_2_, we evaluate the changes simulated in ALL simulations against the changes in the RCP8.5 scenario from the same set of models (Supplementary Table 1). The RCP8.5 simulations are fully coupled and are performed with prescribed increasing CO_2_ from 381 ppm to 936 ppm, as well as changes to other greenhouse gases and aerosol particle emissions from 2006 to 2100. The historical simulations are also used as the control experiments, with CO_2_ increasing from 284 ppm in 1850–379 ppm in 2005. Changes in the RCP8.5 scenario (2081–2100) are calculated relative to the historical period (1986–2005). Daily precipitation in RCP8.5 and historical simulations are used to calculate the changes in monsoon onset and retreat dates and duration. Our ESM projections are consistent with the intensity and monsoon characteristics changes from the same set of models under RCP8.5 scenario (Supplementary Fig. 3 for physical quantity change and Fig. 4 for monsoon timing changes).

### SP-CESM1 simulations

The CMIP5 models use convective parameterisations that can produce biases in the tropics^8,52^. To validate our results with respect to the parameterisations of convection, a convection-permitting model (that is, superparameterization, SP)^53^ is used. In SP-CESM1, the parameterisations of conventional deep and shallow convection are replaced by array-mean convective tendencies from embedded cloud-resolving models (CRMs) that represent unresolved subgrid moist convection explicitly^25,53^. Each grid-column of CAM hosts an independent CRM, configured at kilometre-scale resolution (4 km) in two dimensions with periodic boundaries, which resolves convective-cloud processes on their native scales. The large-scale dynamics are resolved on the outer GCM grid. SP-CESM1 has approximately the same configuration as CESM1-BGC except for the parameterisations of convection and boundary layer processes. The changes in precipitation, ET and precipitation minus ET (P-ET) intensity, as well as monsoon onset and retreat dates and duration in SP-CESM1, are all calculated between 4×CO_2_ (1140 ppm, 2180–2184) and preindustrial periods (285 ppm, 1880–1884; Supplementary Figs. 5–8). Runoff data are not available from SP-CESM1 output. Instead, we compare P-ET change, representing the total changes of runoff and moisture storages, so including components such as soil moisture and water table depth. The comparison between our ESMs and SP-CESM1^25^ is presented in Supplementary Figs. 5–8. The consistency suggests that the large-scale changes in intensity and monsoon characteristics in our CMIP5 models are broadly robust with respect to the representation of convection. We note though, that superparameterization does improve rainfall simulations in the East Asia monsoon region^54,55^.

### Observational datasets

We validate the modelled present-day magnitudes and seasonality of major water component fluxes and vegetation index against observations, and in two independent ways. First, we use field-scale atmospheric (precipitation) and surface fluxes (eddy covariance-based ET) datasets in the East Asia monsoon region to evaluate the performance of the models at field scale. Then, we use observation-based precipitation, ET, LAI and GPP datasets to evaluate model results over all monsoon regions at regionally averaged scales.

### Validation using fluxes observations at field scale

The field-scale precipitation and eddy covariance-based ET data used in the validation are obtained from the global FLUXNET (http://fluxnet.fluxdata.org)^56^, AsiaFlux (http://www.asiaflux.net)^57^ and ChinaFlux (http://www.chinaflux.org/enn/index.aspx)^58^ datasets. To ensure the representativeness of the local climate, the following criteria are used for site selection in our validation. First, at least 3 years of observations are available to remove the impact of interannual variability. Second, due to the coarse model resolution, modelled ET at the land-sea border may be biased in model resample, and so sites at the land-sea border are not included. Third, any site analysed is located within the monsoon domain and not close to the monsoon border. A total of six sites meet the three criteria above, located at Mt. Dinghu (observational periods: 2003–2012), Qianyanzhou (2003–2012), Yucheng (2003–2005), Xishuangbanna (2003–2005), Mt. Changbai (2003–2005) and Duolun grassland (2006–2008). For the ESM data (mean of 1–20 years), the pixels that contained the selected flux sites, values are extracted to compare with observations. In the most general context, there is a broad agreement (Supplementary Fig. 1). We expect some differences at the field scale, due to the scale disagreement between data and the larger grid box scale of ESMs. Also, our ESMs do not include any representation of human water use or management, which may impact on comparisons against large-scale measurements such as evapotranspiration.

### Validation using observational products at regional scale

To avoid the issue of scale disagreement between ESMs and flux sites, and to validate over all monsoon regions, we also obtain observation-based precipitation and ET products from the Climatic Research Unit Time Series (CRU TS) v4.01 (https://crudata.uea.ac.uk/cru/data/hrg/#crurent)^59^ and Global Land Evaporation Amsterdam Model (GLEAM) version 3.3a dataset (https://www.gleam.eu/)^60^, respectively. Observational LAI and GPP are obtained from the Moderate Resolution Imaging Spectroradiometer (MODIS) C6 (10.5067/MODIS/MOD15A2H.006)^61^ and FLUXNET-MTE (http://fluxnet.fluxdata.org)^56^, respectively. The period is from 2000 to 2015, corresponding to CO_2_ ranges of 370–400 ppm in ALL simulations (27–35 years) (Supplementary Figs. 2 and 14).

### Monsoon regions, wet, and dry season definitions

The main land monsoon regions are directly adopted from Wang and Ding^62^. Seven monsoon regions are defined: South Asia, East Asia, Australia, North America, South America, North Africa and South Africa (Fig. 1). The monsoon domains are considered invariant, although we note that altered CO_2_ levels may impact on the spatial characteristics of rainfall patterns^7^. All variables involved in our ESM-based analysis are area-weighted for the model gridboxes in each monsoon region.

Based on monthly precipitation and ET values, averaged over each monsoon region within the light green or light blue boundary on the background map in Fig. 1, the wet season is defined as the months in which ET < P, and vice versa for the dry season (Supplementary Fig. 16). It should be noted that this method gives a much longer wet season (February to December) in the East Asia monsoon region than all other monsoon regions (Supplementary Table 4). Similarly, observational precipitation and ET show that the wet season in East Asia is all year round (January to December) using this method (Supplementary Table 4), possibly due to the rainy winter is this monsoon region. Therefore, in this study the wet season in the East Asia monsoon region is set to May–October as other monsoon regions in the North Hemisphere (South Asia and North America: May to October; North Africa: April to October; Supplementary Fig. 16).

Wet or dry season intensity is the average of monthly precipitation or runoff in corresponding months. Wet and dry season precipitation and runoff changes are determined by changes in both intensity and season length. To accurately calculate the wet and dry season intensity changes, in this context the definition of season lengths is to keep them constant, i.e. based on the historical period for each monsoon region. The units of intensity changes in Fig. 2 are the same as that of annual changes in Fig. 1 – i.e. mm day^−1^, and instead just over different periods (i.e. annual, or wet season or dry season).

### Seasonal characteristics of monsoon

Seasonal characteristics of the monsoon, including monsoon onset and retreat dates and season length require more refined temporal understanding. These two quantities are employed to depict the changes of monsoon characteristics and are based on daily precipitation^7,39^. Three out of four ESMs (CESM1-BGC, IPSL-CM5A-LR and MPI-ESM-LR) are involved in the calculation (Supplementary Table 1), but not NorESM1-ME as this ESM did not provide daily precipitaiton output in the CMIP5 archive. In the calculation, modelled rainfall is first averaged spatially for each of the seven regions, and smoothed temporally, to give a daily climatology of precipitation. Specifically, the daily climatology of precipitation is defined as the sum of the first 12 harmonics of daily average precipitaton. Then, relative climatological mean daily precipitaiton is calculated as the difference between the daily climatology of precipitation and local dry month (January in Northern Hemisphere and July in Southern Hemisphere) mean precipitation. The monsoon onset date is defined as the date when the relative climatological mean daily precipitation first exceeds 5 mm day^−17^ ^39^. Similarly, the retreat date is when relative climatological mean daily precipitation drops below 5 mm day^−1^. Monsoon season length is defined as the difference between retreat and onset dates.

To evaluate the sensitivity of calculated monsoon onset and retreat dates, and so duration, to the precipitation threshold used, we modify the original threshold by −10%, −5%, +5 and 10%. From these new thresholds, we recalculate the monsoon onset and retreat as a sensitivity analysis (Supplementary Fig. 12). A threshold-independent monsoon onset/retreat calculation method, i.e. fractional accumulated precipitation index^43^, is also used to validate our calculated monsoon timings, using the method of Wang and LinHo^39^. In this method, monsoon onset and retreat dates are defined as the dates when the fractional accumulated precipitation first exceeds 0.2 and 0.8, respectively. Fractional accumulated precipitaiton is defined as accumulated precipitation from the start of the year (from January 1 in the Northern Hemisphere and July 1 in the Southern Hemisphere) divided by the total accumulated precipitation at the end of the year. This method is less affected by model wet or dry bias^43^.

### Seasonal characteristics of abundant water-resources period

Diagnostics of ET from ESMs are generally only available at the monthly temporal resolution, and based on these values, we find that most of the ESMs show no changes to both wet and dry season lengths (Supplementary Table 5). However, when considering the more relevant metric of runoff, we instead evaluate the changes in seasonal characteristics of runoff, start date, end date and length of the abundant water-resources period. These estimates follow similar precipitation-based definitions of the monsoon characteristics outlined above. This similarity is expected because daily runoff and precipitation generally exhibit comparable seasonal cycles over all monsoon regions, although peak runoff is slightly delayed compared to peak precipitation in some monsoon regions (Supplementary Fig. 11). Hence daily runoff is first regionally averaged, and smoothed with the sum of first 12 harmonics of daily runoff. Runoff thresholds are calculated as the runoff values at the date corresponding to monsoon onset, based on the historical precipitation analysis above, and for each region (see Supplementary Fig. 17 for runoff thresholds used). The start (end) date of the abundant water-resources period is defined as the date when daily runoff first exceeds (last drops below) the calculated threshold above, and length is defined as their difference. Unfortunately, no daily runoff output is available in the CMIP5 archive for the four ESMs used here. However a similar experiment with one model, CESM1-BGC, which followed the same protocol as the 1pctCO_2_ experiments in CMIP5^40^ (http://kooperman.uga.edu/grl2018/), does have daily runoff and precipitation. These CESM1-BGC simulations differ from CMIP5 by including extended periods of fixed preindustrial and 4×CO_2_ conditions after the 1% per year ramp-up period, the first twenty-years of which are analysed here. These values are adopted in our study, using this one model to calculate the characteristic change of the abundant water resources period (as presented in Fig. 4b). This approach is reasonable because we find that the changes in calculated precipitation-based monsoon onset and retreat dates and related monsoon season length from the CESM1-BGC model (open circles in Fig. 5a) are generally consistent with CMIP5 multi-model mean results (orange bars in Fig. 5a). The larger change in seasonal characteristics for precipitation in the CESM1-BGC simulations, relative to the CMIP5 simulations, may be related in part to a larger relative CO_2_ change during the averaging periods (from fixed 285–1140 ppm in CESM1-BGC and from average 314–1036 ppm in CMIP5) and the fact the climate system had time to adjust to 4 × CO_2_ in CESM1-BGC.

### Moisture budget decomposition

To understand the mechanisms driving precipitation change, precipitation is decomposed into four components, and for both the wet and dry seasons separately. The decomposition is based on the vertical integration of the moisture budget^31^:

1 $$P{^\prime} = - \left\langle {\bar \omega \partial \;_pq{^\prime}} \right\rangle - \left\langle {\omega {^\prime}\partial \;_p\bar q} \right\rangle - \left\langle {{\mathrm{V}} \cdot \nabla q} \right\rangle ^\prime + E{^\prime} + {\mathrm{residual}}.$$

Here the overbar operator – denotes climatology in the historical period and ′ represents change relative to the historical period, and due to raised CO_2_ concentrations. *P*, ω, *q*, **V** and *E* are precipitation, vertical pressure velocity, specific humidity, horizontal velocity and evaporation, respectively. Vertical integral 〈 〉 denotes a mass integration from 1000 hPa to 100 hPa. The five terms on the right-hand side represent the thermodynamic effect, i.e., atmospheric water vapour changes (dWV), altered vertical dynamic effect (dVert), altered horizontal moisture advection (dAdv), land-surface evapotranspiration change (dET) and residual term (Res) as a lower boundary condition.

## Supplementary information

Supplementary Information

## Data Availability

The CMIP5 model outputs (1pctCO2, historical and RCP8.5 scenarios) are freely available from the following locations: https://pcmdi.llnl.gov. The daily runoff data can be accessed through website http://kooperman.uga.edu/grl2018/. SP-CESM1 data are from Mark Branson. Observational water fluxes, ecosystem variables LAI and GPP data are available from the websites in the observational datasets section above.

## Code availability

All computer codes for process and analysis of the data are available from the corresponding author on reasonable request.
