# Supplementary material for: Vegetation forcing modulates global land monsoon and water resources in a CO2-enriched climate
Source: Nat Commun. 2020 Oct 14;11:5184. doi: 10.1038/s41467-020-18992-7 (PMC7560717; doi:10.1038/s41467-020-18992-7)
Supplement: Supplementary file 1 — Supplementary Information [file 41467_2020_18992_MOESM1_ESM.pdf]

Supplementary Information for

Vegetation forcing modulates global land monsoon and water resources in a CO_2-enriched climate

Cui et al.

Supplementary Table 1. Details of the four CMIP5 ESMs used in this study, along with references and data used.

Model information	Model name			
	**CESM1-BGC**	**IPSL-CM5A-LR**	**MPI-ESM-LR**	**NorESM1-ME**
Resolution (lat × lon)	0.9° × 1.25°	1.9° × 3.75°	1.875° × 1.875°	1.9° × 2.5°
Global land mean T/ET	0.53	0.58	0.64	0.50
Stomatal conductance	Ball–Berry[1]	Ball–Berry[1]	Knorr[2]	Ball–Berry[1]
Planetary boundary layer	Holtslag-Boville[3]	Rio-Hourdin[4]	Mailhot-Benoit[5]	Holtslag-Boville[3]
Shallow and deep convection	Hack[6], Zhang-McFarlane[7]	Emanuel[8]	Tiedtke[9]	Hack[6], Zhang-McFarlane[7]
Turbulence closure	Vogelezang-Holtslag[10]	Mellor-Yamada[11]	Lenderink-Holtslag[12]	Vogelezang-Holtslag[10]
Horizontal and vertical diffusions	Boville-Bretherton[13]	Laval[14]	Boville-Bretherton[13]	Boville-Bretherton[13]
References	(Lindsay *et al.*, 2014)[15]	(Dufresne *et al.*, 2013)[16]	(Giorgetta *et al.*, 2013)[2]	(Tjiputra *et al.*, 2013)[17]
Monthly data (including single-forcing, RCP8.5 and historical simulations)	Yes	Yes	Yes	Yes
Daily precipitation (including single-forcing, RCP8.5 and	Yes	Yes	Yes	

historical simulations)	
Daily runoff	Yes*

* Adopted from a similar experiment following the protocol of the carbon-climate feedback experiments in CMIP5 from Kooperman *et al.* (2018) (see Methods).

Supplementary Table 2. List of the three single-forcing CMIP5 simulations used in this study.

**Simulation name**	**CMIP5 terminology**	**Varying CO_2 in atmospheric model**	**Varying CO_2 in land surface model**	**Focus**
ALL	1pctCO_2	Yes	Yes	Both vegetation physiology and climate forcing
VEG	esmFixClim1	No	Yes	Vegetation physiology only
RAD	esmFdbk1	Yes	No	Climate forcing only

Supplementary Table 3. Signs of precipitation, vertical advection and horizontal advection changes in different periods. The symbol "+" or "-" represents whether the change is either positive or negative in sign. The cell with grey background indicates the sign of change is opposite to the sign of mean change of the five regions.

Processes change		Monsoon regions					
		S. Asia	E. Asia	Australia	N. Africa	S. Africa	Five mean
Annual mean	Precipitation	+	+	+	+	+	+
Wet season	Precipitation	+	+	+	+	+	+
	Vertical advection	+	−	+	+	+	+
	Horizontal advection	−	+	+	+	−	+
Dry season	Precipitation	+	+	+	+	−	+
	Vertical advection	+	+	+	+	+	+
	Horizontal advection	−	+	−	−	−	−

Supplementary Table 4. Precipitation minus evapotranspiration (P-ET) and calculated wet and dry seasons in East Asia monsoon region based on observation and 4 model mean data. Months with white (grey) background represent wet (dry) seasons. Wet season is defined as the months where ET < precipitation, and vice versa (see Methods). Observational ET are obtained from the Global Land Evaporation Amsterdam Model (GLEAM) version 3.3a dataset[18,19]. Observational precipitation are derived from Climatic Research Unit Time Series (CRU TS) v4.01[20].

**Month**	**P-ET (mm day^{-1})**	
	**Observation**	**4 model mean (± SD)**
January	0.12	0.13 ± 0.38
February	0.10	0.37 ± 0.29
March	0.08	0.67 ± 0.28
April	0.47	1.48 ± 0.38
May	1.30	1.98 ± 0.67
June	2.52	2.64 ± 0.67
July	2.51	2.30 ± 0.65
August	2.11	2.03 ± 0.44
September	0.96	1.24 ± 0.56
October	0.42	0.30 ± 0.34
November	0.43	0.10 ± 0.08
December	0.23	-0.08 ± 0.22

Supplementary Table 5. Changes in wet and dry season lengths based on monthly precipitation and evapotranspiration (ET). Wet season is defined as the months where ET < precipitation, and vice versa (see Methods). Changes are quantified by the differences of the years 121-140 (CO_2-enriched) of the simulation and the years 1-20 (historical). The unit is month. The length changes in both dry and wet seasons are not significant (*P* > 0.1, *t*-test) over all monsoon regions based on the results of the four ESMs.

**Monsoon region**	**Wet season length change**				**Dry season length change**			
	**CESM1-BGC**	**IPSL-CM5A-LR**	**MPI-ESM-LR**	**NorESM1-ME**	**CESM1-BGC**	**IPSL-CM5A-LR**	**MPI-ESM-LR**	**NorESM1-ME**
S. Asia	0	0	0	1	0	0	0	-1
E. Asia	-1	-3	1	0	1	3	-1	0
Australia	2	0	0	2	-2	0	0	-2
N. America	0	0	-1	-2	0	0	1	2
S. America	1	0	0	0	-1	0	0	0
N. Africa	1	0	0	1	-1	0	0	-1
S. Africa	0	-1	0	0	0	1	0	0

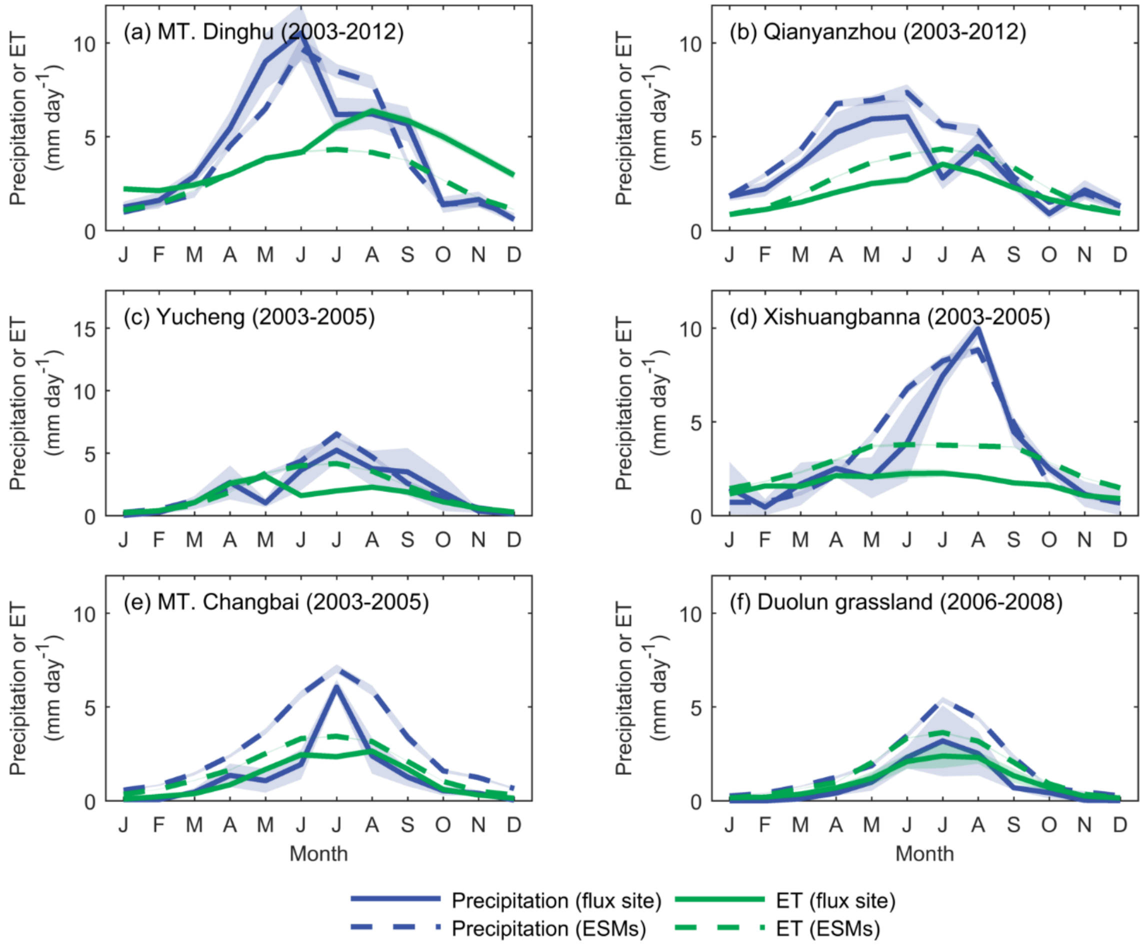

Supplementary Figure 1. Comparisons of monthly precipitation and evapotranspiration (ET) between flux sites and ESMs at the field scale. Modelled values are extracted from the pixel that contained the corresponding flux site. Modelled monthly averages are from the historical periods in ALL simulation (years 1-20). The shaded area indicates standard error of the mean over the periods of observations or simualtions.

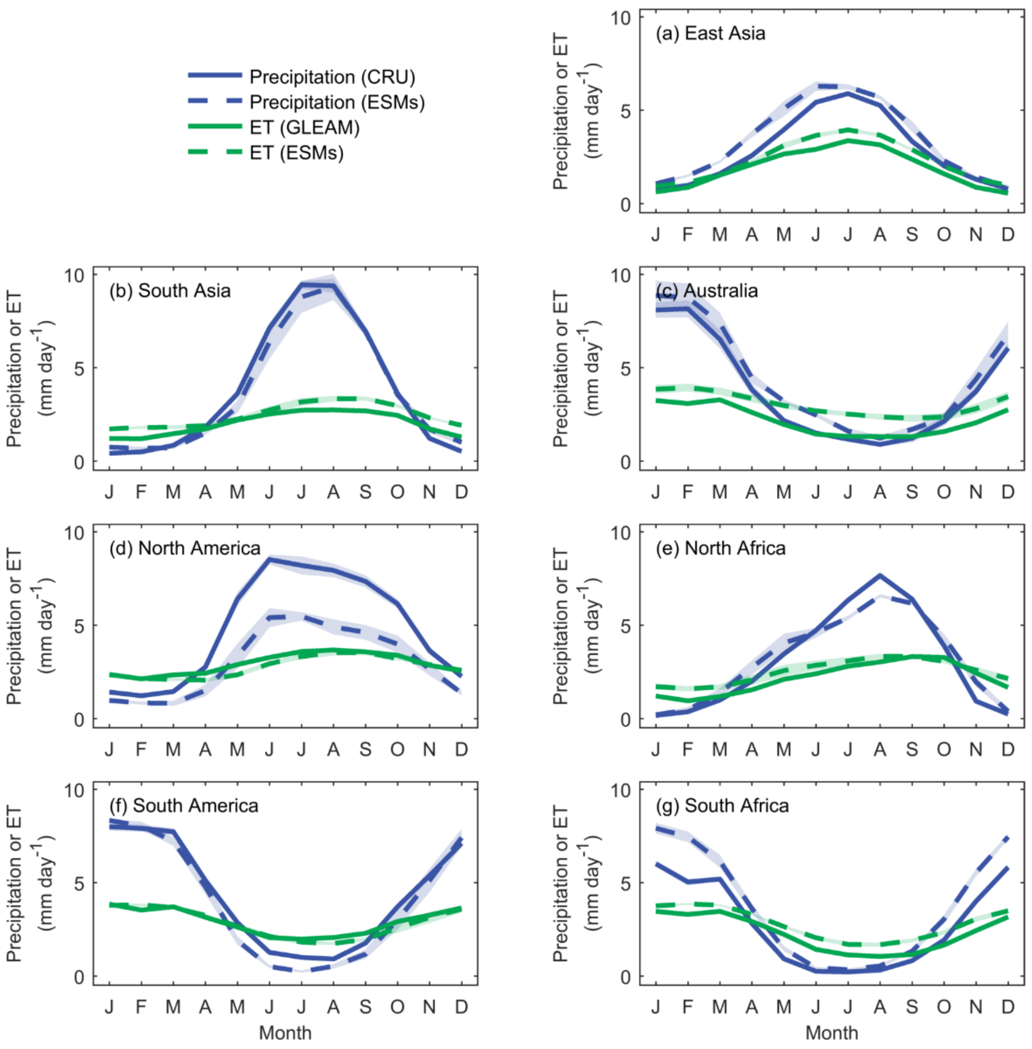

Supplementary Figure 2. Comparisons of monthly precipitation and evapotranspiration (ET) between observation-based products and ESMs at region-averaged scale. The observational period is from year 2000 to 2015 for both precipitation (CRU) and ET (GLEAM), corresponding to CO_2 ranges of 370-400 ppm in ALL simulations (27-35 years). The shaded area indicates standard error of the mean over the periods of observations or simualtions. Monsoon region as marked.

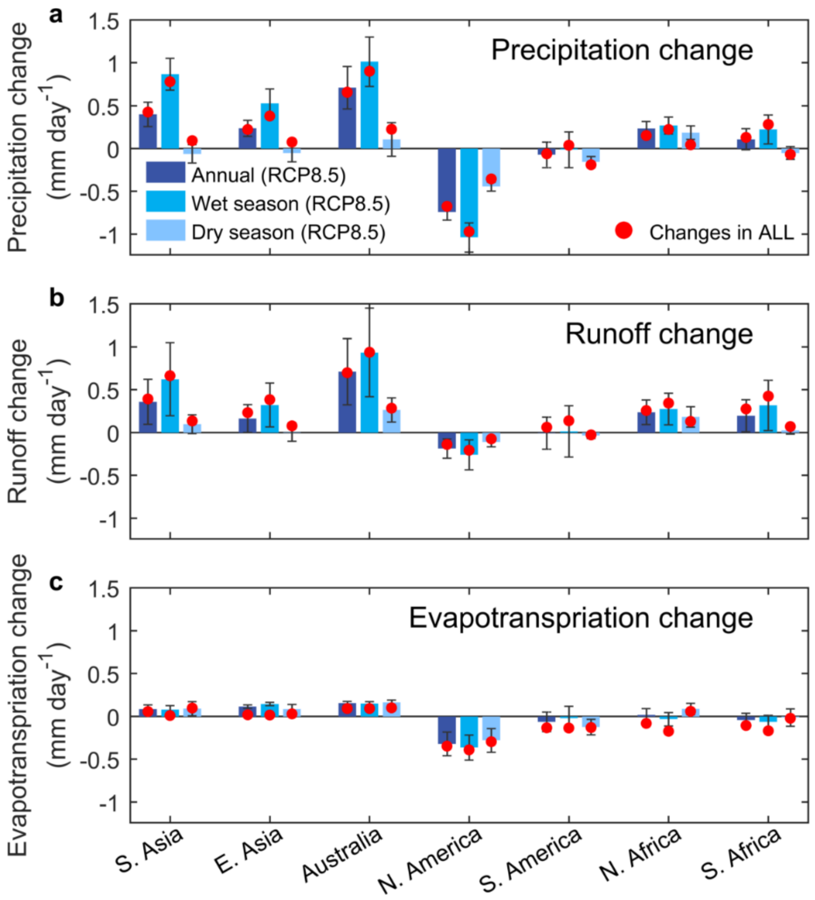

Supplementary Figure 3. Comparisons of changes in precipitation, runoff and evapotranspiration (ET) between ALL simulations and RCP8.5 scenario. Mean changes in the RCP8.5 scenario (2081-2100) are calculated relative to the historical period (1986-2005). The error bars indicate standard errors of ESM means over the 20-yr simulations in RCP8.5. The red dots are the mean changes (i.e. across four ESMs) in our ALL forcings 4×CO_2 simulations (years 121-140) and historical simulations (years 1-20).

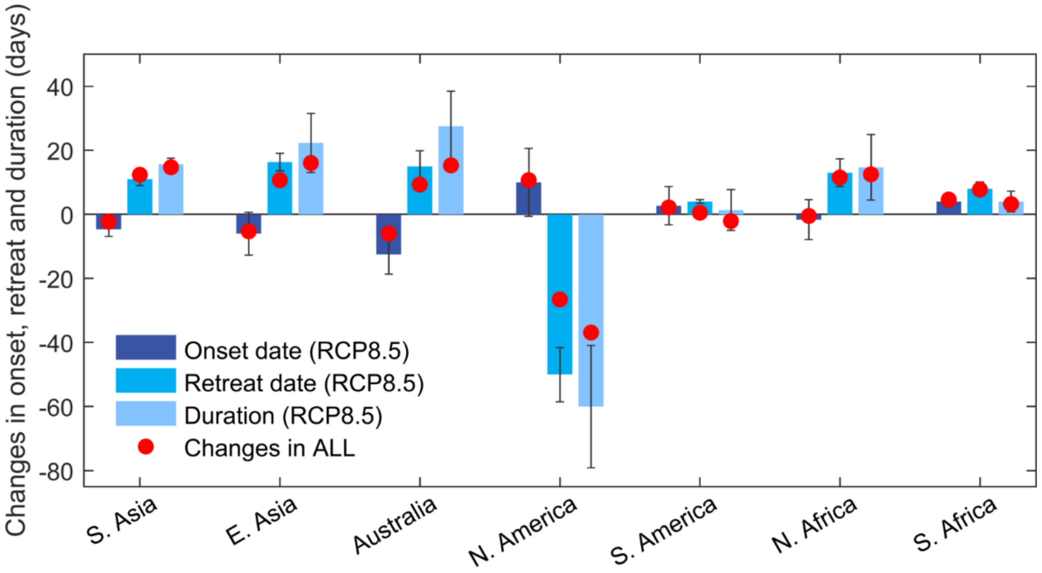

Supplementary Figure 4. Comparisons of changes in monsoon onset, retreat dates and duration between ALL simulations and RCP8.5 scenario. Mean changes in the RCP8.5 scenario (2081-2100) are calculated relative to the historical period (1986-2005). The error bars indicate standard errors of the mean of the three ESMs used. The red dots are mean changes (i.e. across four ESMs) in our ALL forcings 4×CO_2 simulations (years 121-140) and historical simulations (years 1-20).

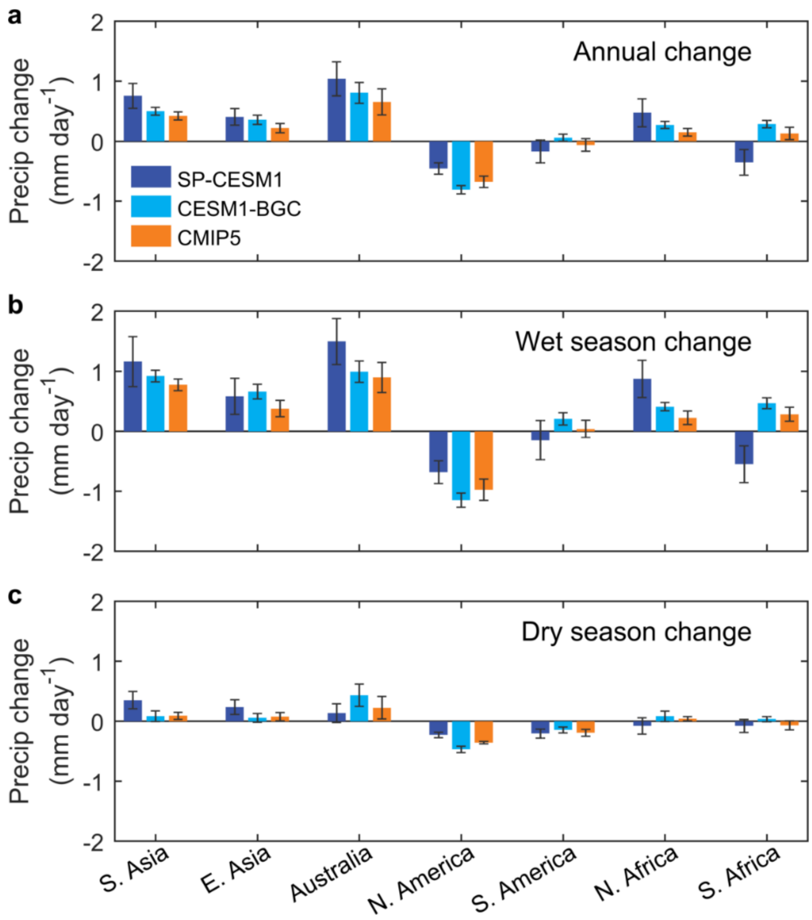

Supplementary Figure 5. Comparison of changes in annual, dry and wet season precipitation between SP-CESM1, CESM1-BGC (CMIP5) and CMIP5 4 ESMs mean. Changes in the convection-resolving model SP-CESM1 are calculated between 4×CO_2 (2180-2184, 1140 ppm) and pre-industrial periods (1880-1884, 285 ppm). Changes in the mean of CMIP5 ESMs are calculated between 4×CO_2 (121-140 model years, 1036 ppm) and historical periods (1-20 model years, 314 ppm). Error bars indicate standard errors of the mean over the periods of simulations.

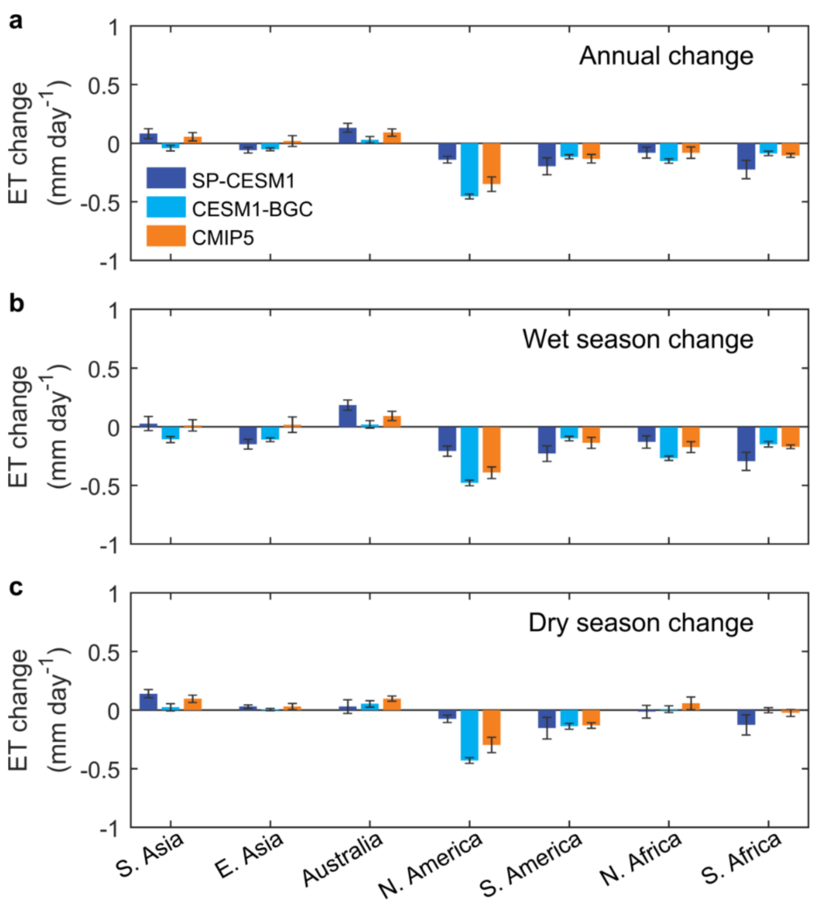

Supplementary Figure 6. Comparison of changes in annual, dry and wet season evapotranspiration (ET) between SP-CESM1, CESM1-BGC (CMIP5) and CMIP5 4 ESMs mean. Changes in the convection-resolving model SP-CESM1 are calculated between 4×CO_2 (2180-2184, 1140 ppm) and pre-industrial periods (1880-1884, 285 ppm). Changes in the mean of CMIP5 ESMs are calculated between 4×CO_2 (121-140 model years, 1036 ppm) and historical periods (1-20 model years, 314 ppm). Error bars indicate standard errors of the mean over the periods of simulations.

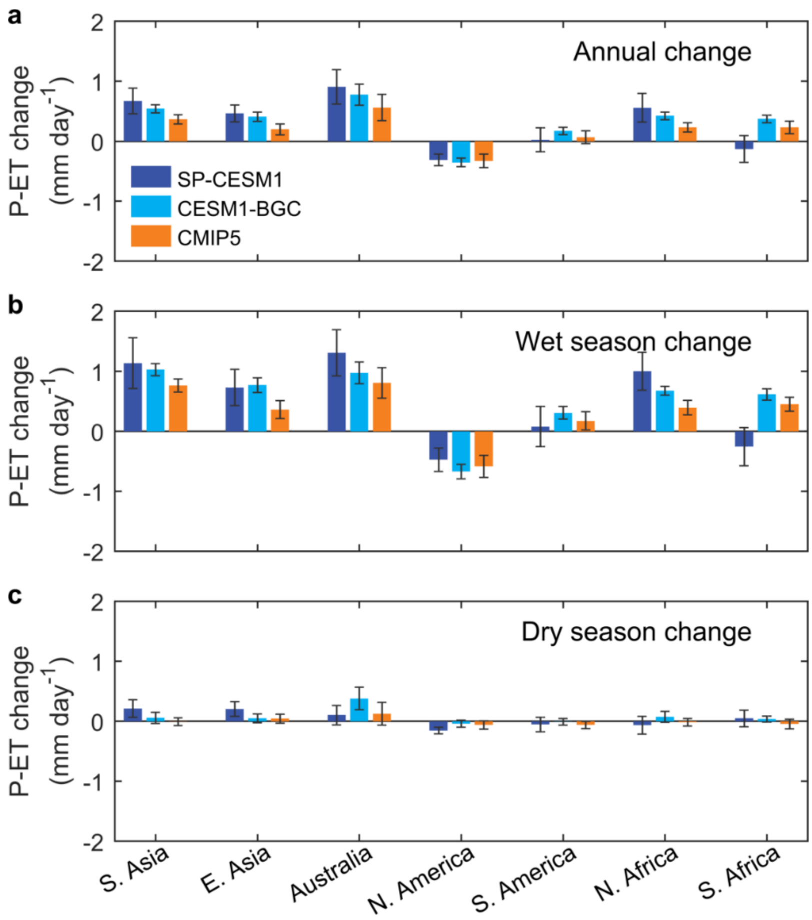

Supplementary Figure 7. Comparison of changes in annual, dry and wet season precipitation minus evapotranspiration (P-ET) between SP-CESM1, CESM1-BGC (CMIP5) and CMIP5 4 ESMs mean. Changes in the convection-resolving model SP-CESM1 are calculated between 4×CO_2 (2180-2184, 1140 ppm) and pre-industrial periods (1880-1884, 285 ppm). Changes in the mean of CMIP5 ESMs are calculated between 4×CO_2 (121-140 model years, 1036 ppm) and historical periods (1-20 model years, 314 ppm). Error bars indicate standard errors of the mean over the periods of simulations.

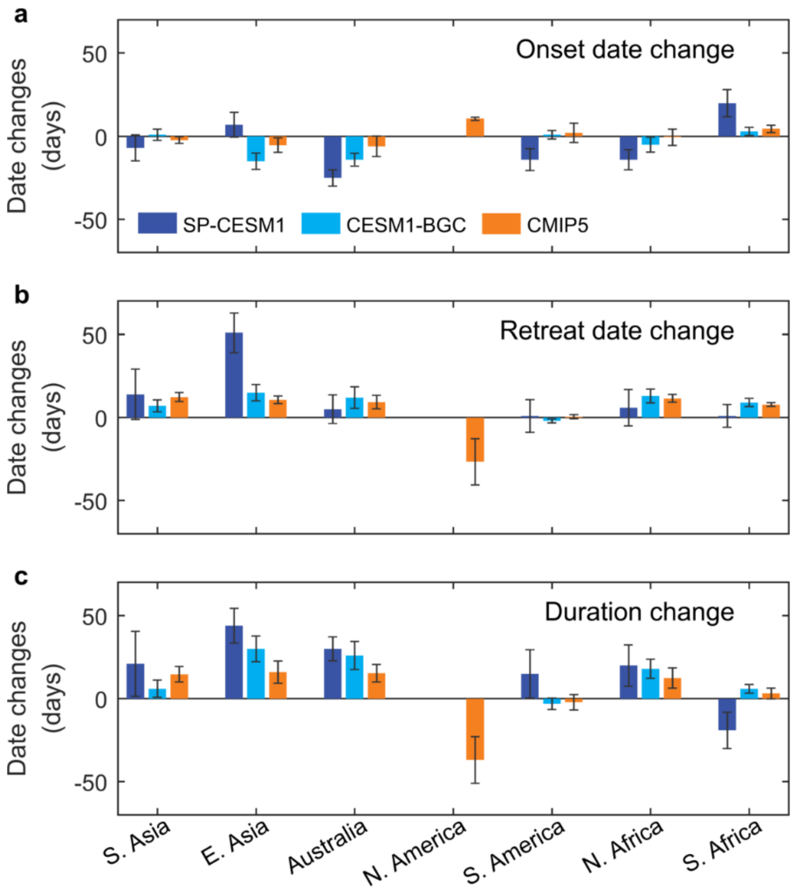

Supplementary Figure 8. Comparisons of changes in monsoon onset, retreat dates and duration between SP-CESM1, CESM1-BGC (CMIP5) and CMIP5 4 ESMs mean. Changes in the convection-resolving model SP-CESM1 are calculated between 4×CO_2 (2180-2184, 1140 ppm) and pre-industrial periods (1880-1884, 285 ppm). Changes in the mean of CMIP5 ESMs are calculated between 4×CO_2 (121-140 model years, 1036 ppm) and historical periods (1-20 model years, 314 ppm). Error bars indicate standard errors of the mean over the periods of simulations. The changes in North America from SP-CESM1 and CESM1-BGC are not available due to model dry bias of CESM1-BGC (Supplementary Fig. 2d) and significant precipitation reductions at 4×CO_2 (Supplementary Fig. 11d).

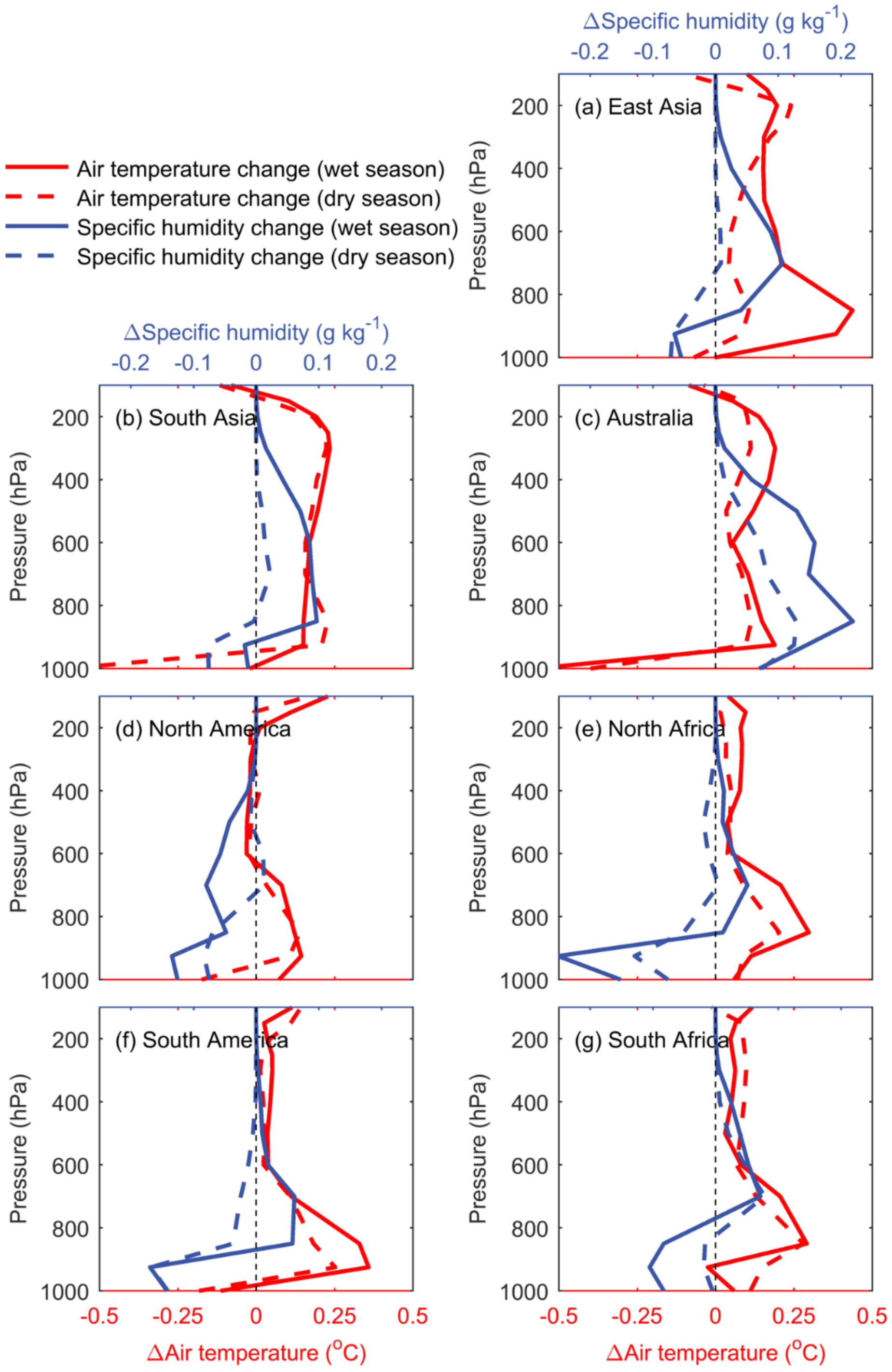

Supplementary Figure 9. Changes in multi-model mean vertical profiles of air temperature and specific humidity for wet and dry seasons in the physiological (VEG) simulations. The changes are quantified by the differences of the years 121-140 (approximately 4 × CO_2-enriched) of the simulation minus the years 1-20 (historical).

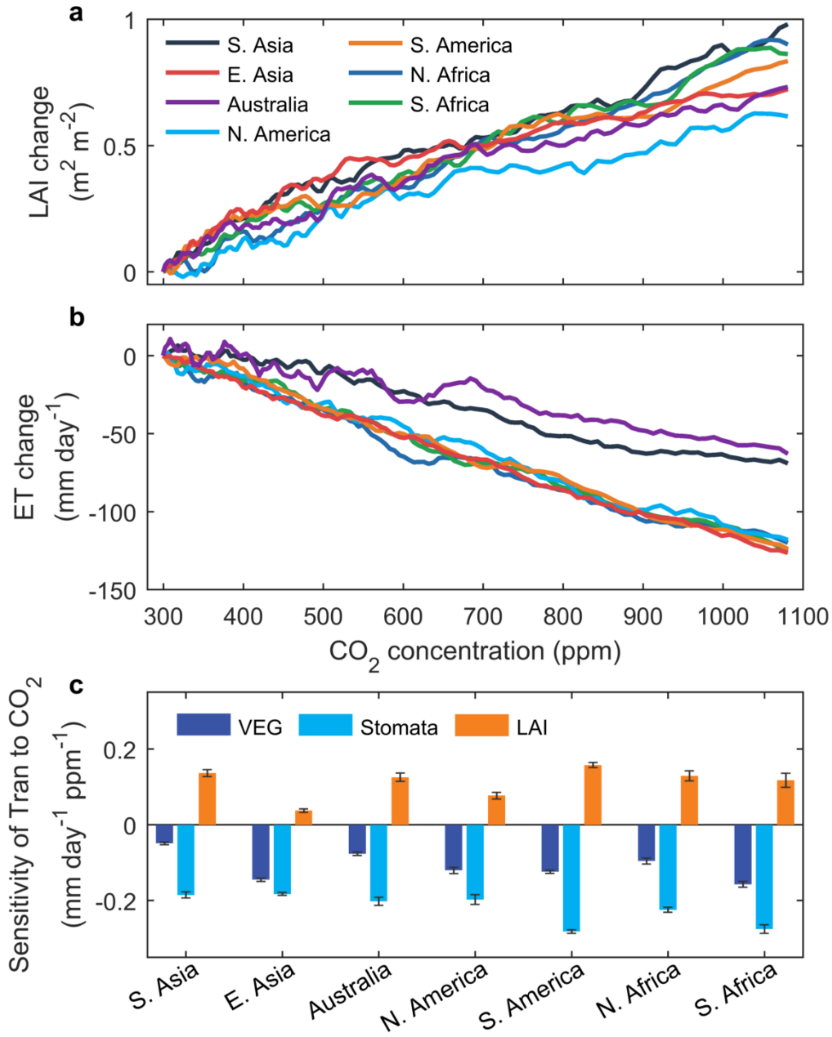

Supplementary Figure 10. Change in anomalies of leaf area index (LAI), evapotranspiration (ET) and transpiration with CO_2 in physiological (VEG) simulations. **a-b**, Changes in anomalies of multi-model mean **a**) LAI and **b**) ET with CO_2 over all monsoon regions in VEG simulations. **c**, Sensitivity of transpiration to rising CO_2 in the last 20-year period of VEG simulations (years 121-140) (dark blue bars). In panel **c**, the response of transpiration to rising CO_2 is further partitioned into the parts caused by stomatal closure (light blue bars) and LAI increase (orange bars) (see Methods). The sensitivity is estimated by a linear regression between the transpiration and CO_2 concentration. The error bars indicate standard errors of the mean of the four ESMs.

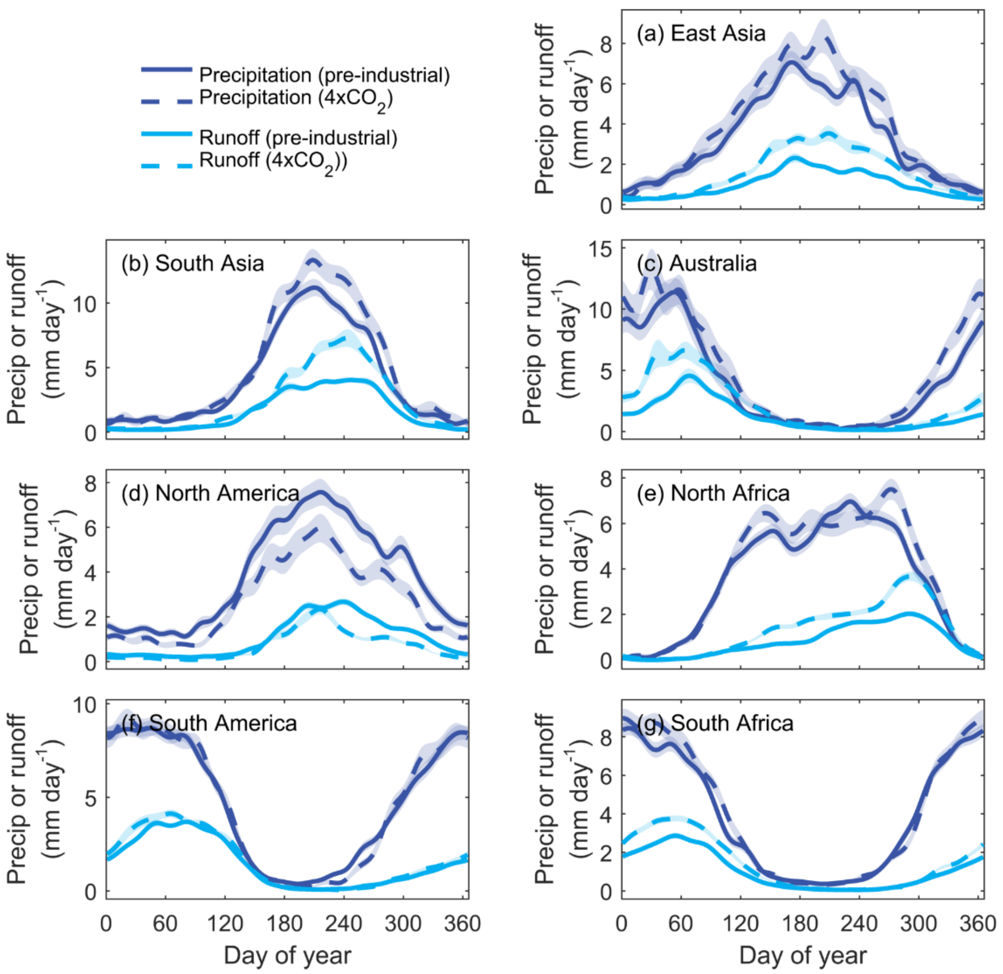

Supplementary Figure 11. Seasonal cycles of daily precipitation and runoff in pre-industrial and 4×CO_2 periods. Shown are the 20-year means in pre-industrial and 4×CO_2 ALL simulations and from the CESM1-BGC climate model. The daily precipitation and runoff are smoothed with the sum of the first 12 harmonics of daily values. The shaded area indicates standard error of the mean precipitation or runoff over the 20-year periods.

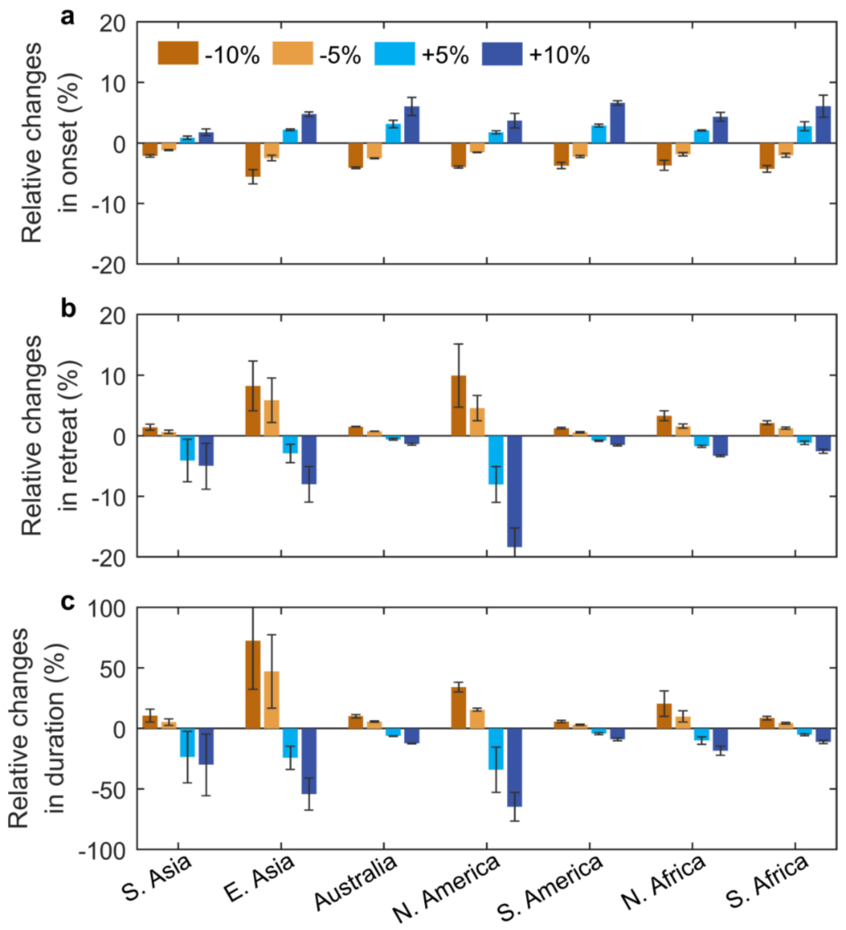

Supplementary Figure 12. Sensitivity analysis of monsoon onset, retreat dates and duration to different precipitation thresholds. Relative changes in monsoon onset **a**), retreat **b**) and duration **c**) in response to -10%, -5%, +5% and +10% changes of precipitation threshold (5 mm day^{-1}) as widely used in previous studies[21,22]. The error bars are standard errors of the mean of the three ESMs with daily precipitation. The sensitivity test is conducted for the historical period (1-20 model years) and in the ALL simulation.

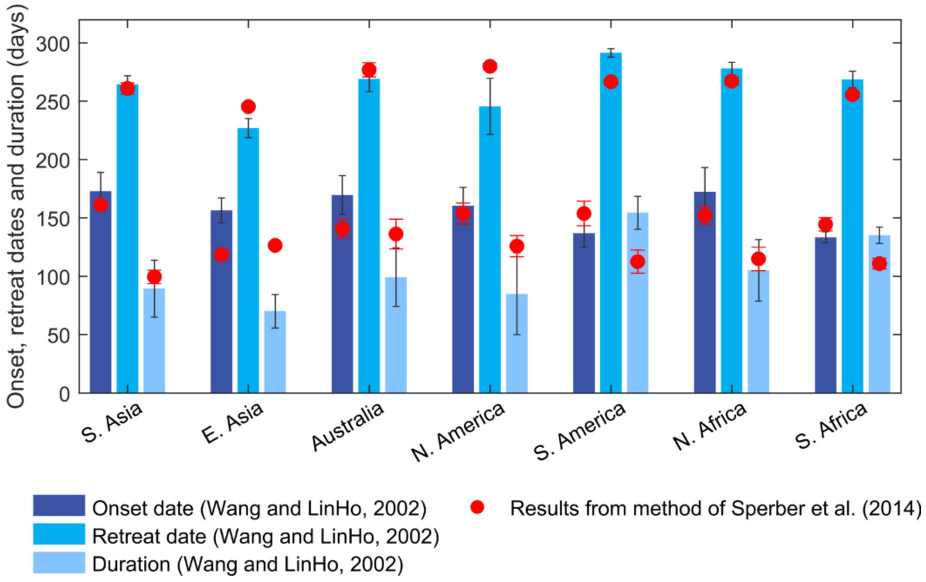

Supplementary Figure 13. Comparison of monsoon onset, retreat dates and duration calculated by two different methodologies. The bars indicate results from the precipitation threshold-based method of Wang and LinHo [22]. The red points are results from the fractional accumulated precipitation-based method of Sperber and Annamalai [23]. The error bars are standard errors of the mean of the three ESMs. The comparison is conducted in the historical period (1-20 model years) of the ALL simulation. Note the onset and retreat dates of monsoon regions in the Southern Hemisphere are counted from 1st June for easy comparison with other regions.

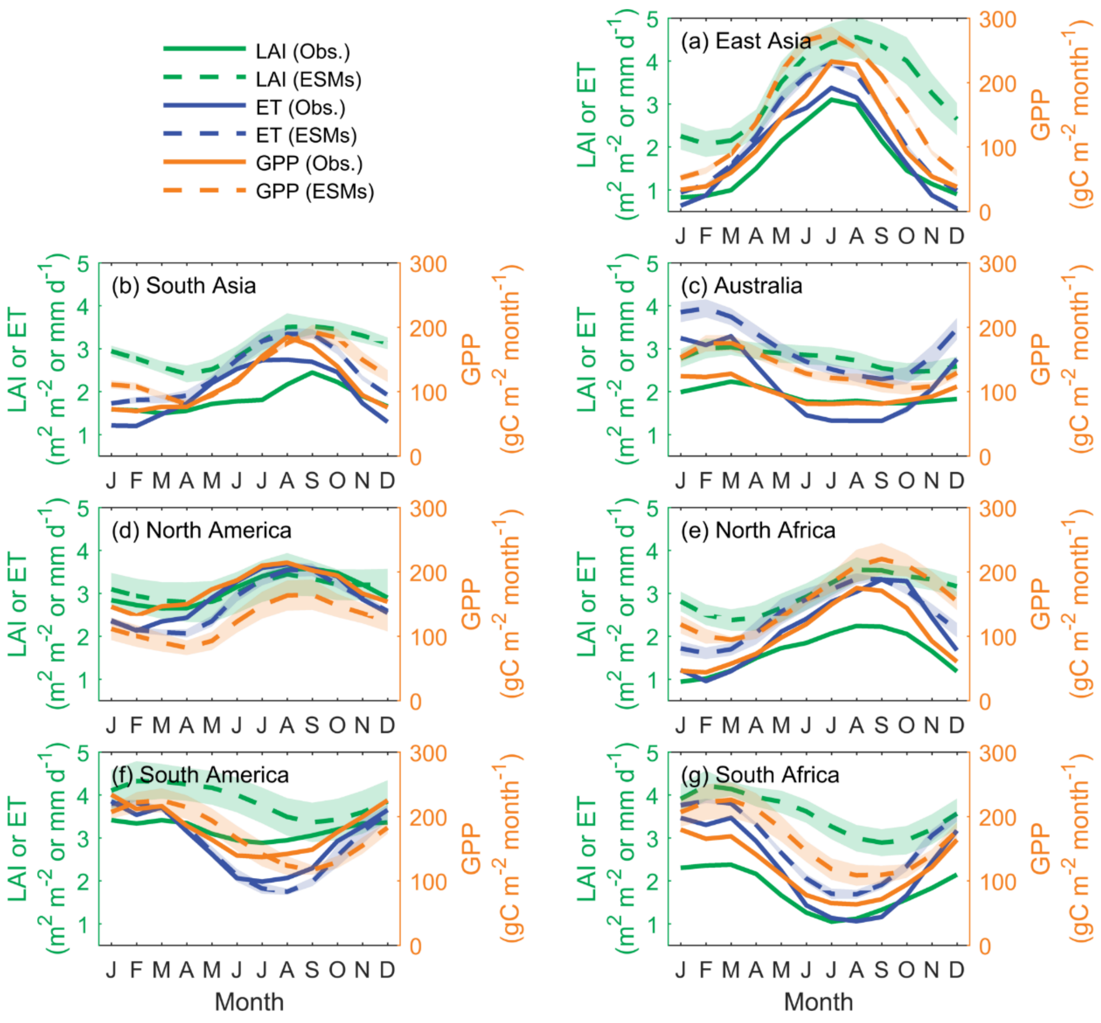

Supplementary Figure 14. Comparisons of monthly leaf area index (LAI), gross primary productivity (GPP) and evapotranspiration (ET) between observations and ESMs at region-averaged scale. Presented are observational monthly mean values of LAI, ET and GPP, obtained from MODIS C6 (2000-2015), GLEAM v3.3a (2000-2015) and MTE upscaling from FLUXNET (2000-2011), respectively. Modelled results are from 27-35 years or 27-33 years in ALL simulations, corresponding to the CO_2 ranges of years 2000-2015 or 2000-2011. The shaded area indicates standard error of the mean over the observed or simulated periods.

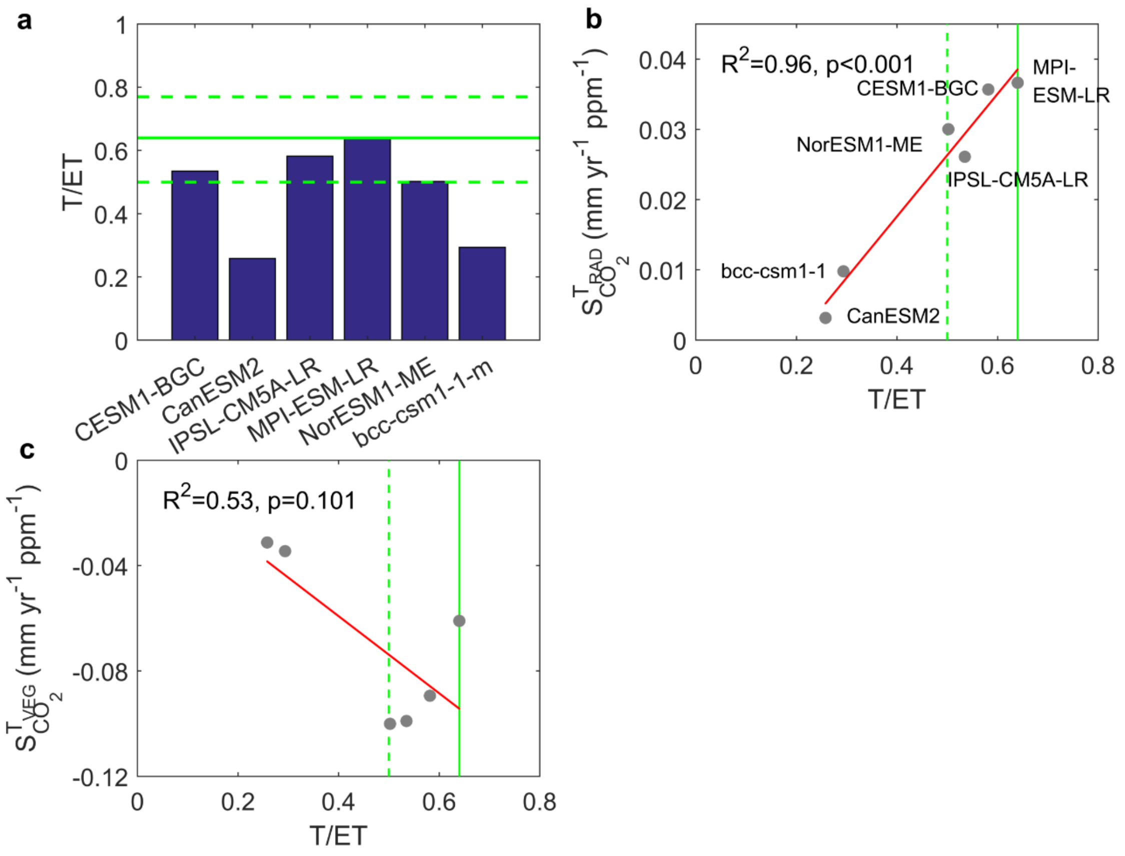

Supplementary Figure 15. Relationship between the ratio of global land mean transpiration to total evapotranspiration (T/ET) of ESM and the sensitivity of water cycle component to rising CO_2 concentration. a, Comparison of global land mean T/ET from six ESMs (blue bars). Green solid lines denote global land mean T/ET, while green dashed lines indicate one standard deviations from isotope-based observational results of Good *et al.* (2015)[24] (and similarly in **b-c**). **b**, Relationship between global land mean T/ET and sensitivity of transpiration to CO_2 radiative (RAD) forcing ($S_{CO_2}^{T_{RAD}}$) for the six ESMs (read line). The sensitivity is estimated by a linear regression between the transpiration and CO_2 concentration in RAD simulation. **c**, Relationship between global land mean T/ET and the sensitivity of transpiration to direct CO_2 physiological (VEG) forcing ($S_{CO_2}^{T_{PHY}}$) for the six ESMs (red line). The sensitivity is again estimated by a linear regression, but now between the transpiration and CO_2 concentration in VEG simulation.

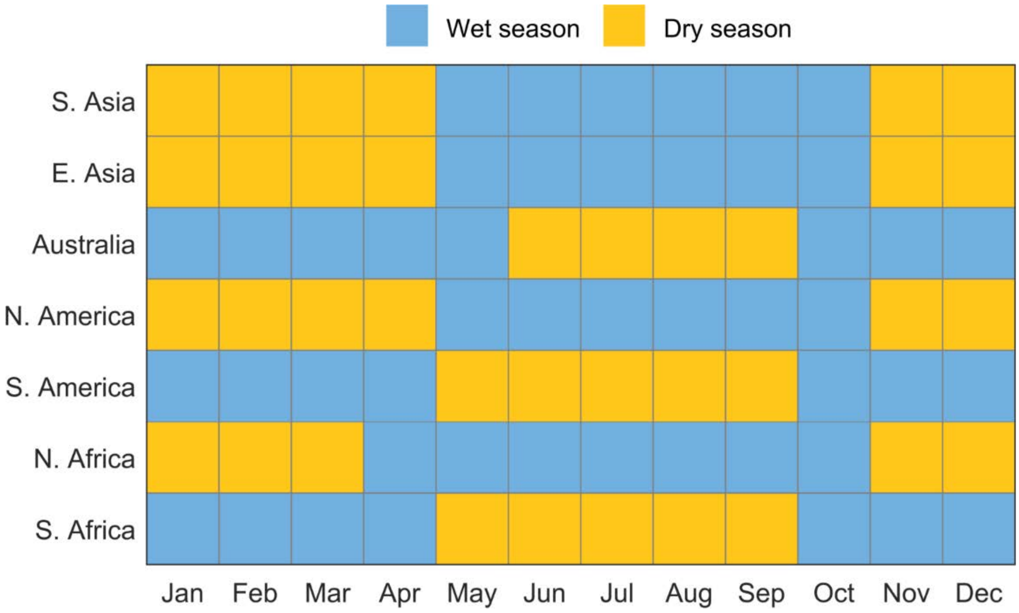

Supplementary Figure 16. Wet and dry season for each monsoon region. Monthly precipitation and evapotranspiration (ET) values are first averaged over each monsoon region within the boundary in Fig. 1. The wet season is defined as the months where ET < precipitation, and vice versa for the dry season (see Methods).

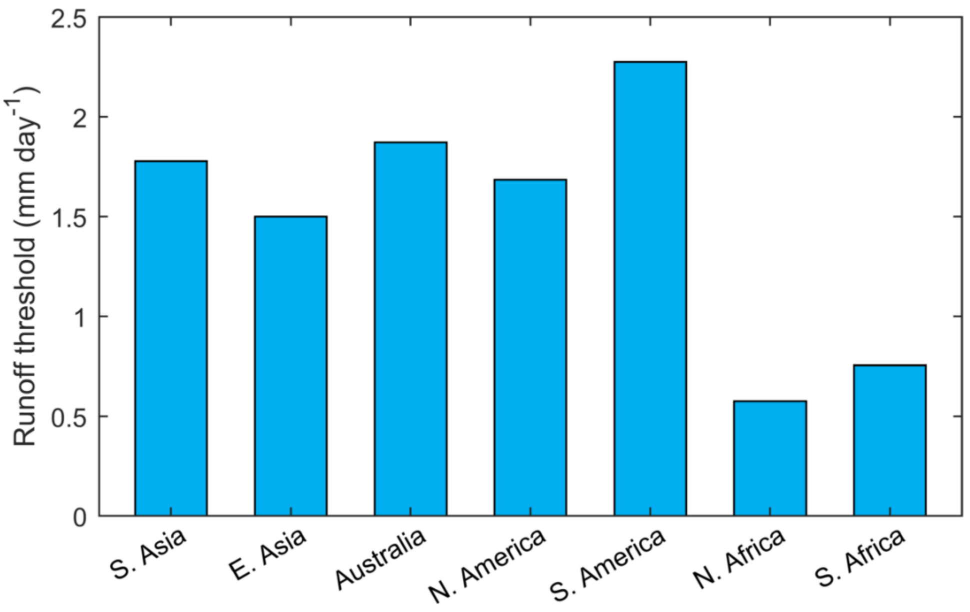

Supplementary Figure 17. Runoff threshold used to calculate abundant water-resources period for each monsoon region. Runoff thresholds are calculated as the runoff values at the date corresponding to monsoon onset date, as based on the historical precipitation analysis (see Methods for details).

Supplementary References

1. Ball, J. T., Woodrow, I. E. & Berry, J. A. in *Progress in Photosynthesis Research: Volume 4 Proceedings of the VIIth International Congress on Photosynthesis Providence, Rhode Island, USA, August 10–15, 1986* (ed J. Biggins) 221-224 (Springer Netherlands, 1987).
2. Giorgetta, M. A. *et al.* Climate and carbon cycle changes from 1850 to 2100 in MPI-ESM simulations for the Coupled Model Intercomparison Project phase 5. *J. Adv. Model Earth Syst.* **5**, 572-597 (2013).
3. Holtslag, A. A. M. & Boville, B. A. Local Versus Nonlocal Boundary-Layer Diffusion in a Global Climate Model. **6**, 1825-1842 (1993).
4. Rio, C. & Hourdin, F. A Thermal Plume Model for the Convective Boundary Layer: Representation of Cumulus Clouds. **65**, 407-425 (2008).
5. Mailhot, J. & Benoit, R. A Finite-Element Model of the Atmospheric Boundary Layer Suitable for Use with Numerical Weather Prediction Models. **39**, 2249-2266 (1982).
6. Hack, J. J. Parameterization of moist convection in the National Center for Atmospheric Research community climate model (CCM2). **99**, 5551-5568 (1994).
7. Zhang, G. J. & McFarlane, N. A. Sensitivity of climate simulations to the parameterization of cumulus convection in the Canadian climate centre general circulation model. *Atmos. Ocean* **33**, 407-446 (1995).
8. Emanuel, K. A. A Scheme for Representing Cumulus Convection in Large-Scale Models. **48**, 2313-2329 (1991).
9. Tiedtke, M. A Comprehensive Mass Flux Scheme for Cumulus Parameterization in Large-Scale Models. **117**, 1779-1800 (1989).
10. Vogelezang, D. H. P. & Holtslag, A. A. M. Evaluation and model impacts of alternative boundary-layer height formulations. *Boundary Layer Meteorol.* **81**, 245-269 (1996).
11. Mellor, G. L. & Yamada, T. A Hierarchy of Turbulence Closure Models for Planetary Boundary Layers. **31**, 1791-1806 (1974).

12 Lenderink, G. & Holtslag, A. A. M. Evaluation of the kinetic energy approach for modeling turbulent fluxes in stratocumulus. *Monthly Weather Review* **128**, 244-258 (2000).

13 Boville, B. A. & Bretherton, C. S. Heating and Kinetic Energy Dissipation in the NCAR Community Atmosphere Model. **16**, 3877-3887 (2003).

14 Laval, K., Sadourny, R. & Serafini, Y. Land surface processes in a simplified general circulation model. *Geophysical & Astrophysical Fluid Dynamics* **17**, 129-150 (1981).

15 Lindsay, K. *et al.* Preindustrial-Control and Twentieth-Century Carbon Cycle Experiments with the Earth System Model CESM1(BGC). *J. Climate* **27**, 8981-9005 (2014).

16 Dufresne, J.-L. *et al.* Climate change projections using the IPSL-CM5 Earth System Model: from CMIP3 to CMIP5. *Clim. Dyn.* **40**, 2123-2165 (2013).

17 Tjiputra, J. F. *et al.* Evaluation of the carbon cycle components in the Norwegian Earth System Model (NorESM). *Geoscientific Model Development* **6**, 301-325 (2013).

18 Martens, B. *et al.* GLEAM v3: satellite-based land evaporation and root-zone soil moisture. *Geoscientific Model Development* **10**, 1903-1925 (2017).

19 Miralles, D. G. *et al.* Global land-surface evaporation estimated from satellite-based observations. *Hydrol. Earth Syst. Sc.* **15**, 453-469 (2011).

20 Harris, I., Jones, P. D., Osborn, T. J. & Lister, D. H. Updated high-resolution grids of monthly climatic observations - the CRU TS3.10 Dataset. *Int. J. Climatol.* **34**, 623-642 (2014).

21 Kitoh, A. *et al.* Monsoons in a changing world: A regional perspective in a global context. *J. Geophys. Res. Atmos.* **118**, 3053-3065 (2013).

22 Wang, B. & LinHo. Rainy season of the Asian–Pacific summer monsoon. *J. Climate* **15**, 386-398 (2002).

23 Sperber, K. R. & Annamalai, H. The use of fractional accumulated precipitation for the evaluation of the annual cycle of monsoons. *Clim. Dyn.* **43**, 3219-3244 (2014).

24 Good, S. P., Noone, D. & Bowen, G. Hydrologic connectivity constrains partitioning of global terrestrial water fluxes. *Science* **349**, 175-177 (2015).
